# Rice *UBC13*, a candidate housekeeping gene, is required for K63-linked polyubiquitination and tolerance to DNA damage

**DOI:** 10.1186/1939-8433-5-24

**Published:** 2012-09-08

**Authors:** Yuepeng Zang, Qian Wang, Chenyu Xue, Mengnan Li, Rui Wen, Wei Xiao

**Affiliations:** 1 College of Life Sciences, Capital Normal University, Beijing, China 100048; 2 Department of Microbiology and Immunology, University of Saskatchewan, Saskatoon, SK Canada S7N 5E5

**Keywords:** Rice, Ubc13, K63-linked polyubiquitination, DNA-damage tolerance, Signal transduction

## Abstract

While plant growth and reproduction is dependent on sunlight, UV irradiation from sunlight is one of the major genotoxic stresses that threaten plant survival and genome stability. In addition, many environmental chemicals can also damage the plant genome. In yeast and mammalian cells protection against the above genome instability is provided by an error-free DNA-damage tolerance (DDT) pathway, which is dependent on Ubc13-mediated K63-linked polyubiquitination of the proliferating cell nuclear antigen (PCNA). In this study, we isolated the *UBC13* gene from rice and characterized its functions. Expression of *OsUBC13* can protect a yeast *ubc13* null mutant against spontaneous and environmental DNA damage. Furthermore, OsUbc13 physically interacts with human Ubc13 partners Mms2 and Uev1A, and catalyzes K63 polyubiquitination *in vitro*. These observations collectively suggest that the K63 polyubiquitination is conserved in rice, and that *OsUBC13* may be involved in DDT and other cellular processes. In addition, *OsUBC13* is constitutively expressed at a high level even under various stress conditions, suggesting that it is a housekeeping gene.

**Electronic supplementary material:**

The online version of this article (doi:10.1186/1939-8433-5-24) contains supplementary material, which is available to authorized users.

## Background

Ubiquitination is an essential protein posttranslational modification (PTM) process found in all eukaryotic organisms. This modification by a 76 amino acid ubiquitin (Ub) determines protein turnover, regulation and molecular function, and provides a complex regulation of various biological processes ([Hershko and Ciechanover 1998]). The most well-understood ubiquitination pathways are the ubiquitin-proteasome system (UPS) that mediates protein degradation ([Hochstrasser 1996]). UPS has been reported to be involved in almost all aspects of plant biology, including phytohormone signalling (auxin, gibberellins, jasmonic acid etc.), plant morphogenesis, immune response, self-incompatibility, chromatin modification and epigenetic regulation ([Vierstra 2009]). Ubiquitination, as an enzymatic cascade, is catalyzed by members of three enzyme families: ubiquitin-activating enzyme (Uba or E1), ubiquitin-conjugating enzyme (Ubc or E2) and ubiquitin ligase (Ubl or E3) enzymes. Before conjugation takes place, the initial activation of ubiquitin is performed by E1 in an ATP-dependent manner. The activated ubiquitin is then transferred from E1 to an active-site cysteine of E2 via a trans-esterification reaction. Finally, a Ub-loaded E2 selectively interacts with a cognate E3 that recruits a specific substrate. The C-terminal glycine of Ub is linked to the ϵ-amino group lysine residue of either the target protein or a previously attached Ub ([Hershko and Ciechanover 1998]; van [Wijk and Timmers 2009]). Unlike HECT (homologous to E6-AP C terminus) E3s that first accept the activated Ub prior to substrate modification ([Rotin and Kumar 2009]), RING (really interesting new gene) and U-box E3s act as a platform and provide an optimal conformation for the Ub transfer while binding both the substrate and Ub-loaded E2 ([Ardley and Robinson 2005]; [Deshaies and Joazeiro 2009]).

In this enzymatic cascade, the highly-conserved E2 family that couples the activation of Ub to the conjugation event appears to play a central role. E2s directly influence which surface lysine residue in Ub will be attached to the incoming Ub, which affects the destiny of the substrate (Burroughs et al. [2008]; [Pickart 2000]; [Pickart and Fushman 2004]). In the UPS pathway, the target protein is modified by a Lys48 (K48)-linked poly-Ub chain that is recognized and degraded by 26 S proteasome ([Hershko and Ciechanover 1998]). In Arabidopsis this process is catalyzed by at least 37 E2s and accompanied by potentially over 1,400 different E3s to recognize different substrates in various biological pathways (Kraft et al. [2005]; [Vierstra 2009]). In contrast, only one E2, Ubc13, has been reported to be capable of catalyzing K63-linked poly-Ub chain assembly in eukaryotes, from unicellular yeasts to human, and this non-canonical poly-Ub chain assembly requires a heterodimeric E2 complex consisting of Ubc13 and a Ubc-E2 variant (Uev) ([Hofmann and Pickart 1999]; McKenna et al. [2001]). In *Saccharomyces cerevisiae*, Ubc13-Uev (Mms2) complex coupled with the E3 Rad5 modifies proliferating cell nuclear antigen (PCNA) at its K164 residue with K63-linked poly-Ub chain, which promotes error-free DNA-damage tolerance (DDT, also known as post-replication repair, PRR) as one of two means of bypassing replication-blocking lesions (Broomfield et al. [1998]; Hoege et al. [2002]; [Hofmann and Pickart 1999]; [Ulrich and Jentsch 2000]). Another lesion bypass method is called translesion DNA synthesis (TLS), which utilizes non-essential DNA polymerases including Polη (Rev3 + Rev7), Polζ, and Rev1 ([Ulrich and Jentsch 2000]; Xiao et al. [2000]). It is now clear that the K164 residue of PCNA is monoubiquitinated by the E2-E3 complex Rad6-Rad18, which promotes TLS, whereas its sequential K63-linked polyubiquitination by Mms2-Ubc13-Rad5 promotes error-free DDT (Hoege et al. [2002]; [Pastushok and Xiao 2004]). Interestingly, the PCNA-K164 residue can also be SUMOylated by the E2-E3 complex Siz1-Ubc9 (Hoege et al. [2002]; [Stelter and Ulrich 2003]). Consequently, the SUMOylated PCNA can recruit the DNA helicase Srs2 to stalled replication forks to prevent unwanted homologous recombination (Papouli et al. [2005]; Pfander et al. [2005]).

Plants, as sessile lifestyle organisms, have to face numerous environmental stresses including genotoxic stress, such as UV irradiation and chemical pollutants. This wide range of stresses threatens plants survival, reduces crop yields and food quality, and compromises world food security. Although there are some reports on genes involved in plant DDT, most describe TLS polymerases (Anderson et al. [2008]; [Curtis and Hays 2007]; Garcia-Ortiz et al. [2004]; Sakamoto et al. [2003]; Takahashi et al. [2005]) and a few on error-free DDT genes from Arabidopsis (Chen et al. [2008]; Wen et al. [2006]; Wen et al. [2008]), with little elucidation on the mechanism. Surprisingly, to the best of our knowledge, there is no report on the isolation and characterization of genes involved in DDT in crop plants. In this study, the rice *UBC13* gene was isolated and functionally characterized. It was found that *OsUBC13* is able to functionally complement the yeast *ubc13* null mutant and that OsUbc13 protein can interact with Uevs from other species and mediate K63-linked polyubiquitination in vitro, suggesting that it may play similar roles in rice. It was also found that both the transcript and protein levels of OsUbc13 remain stable during plant growth and development, as well as facing various biotic and abiotic stresses. We argue that *OsUBC13* is a candidate housekeeping gene and its expression may be used as a useful internal control for the study of other rice genes or gene products.

## Results

### Identification and sequence analysis of the *OsUBC13* gene

**Figure 1:**
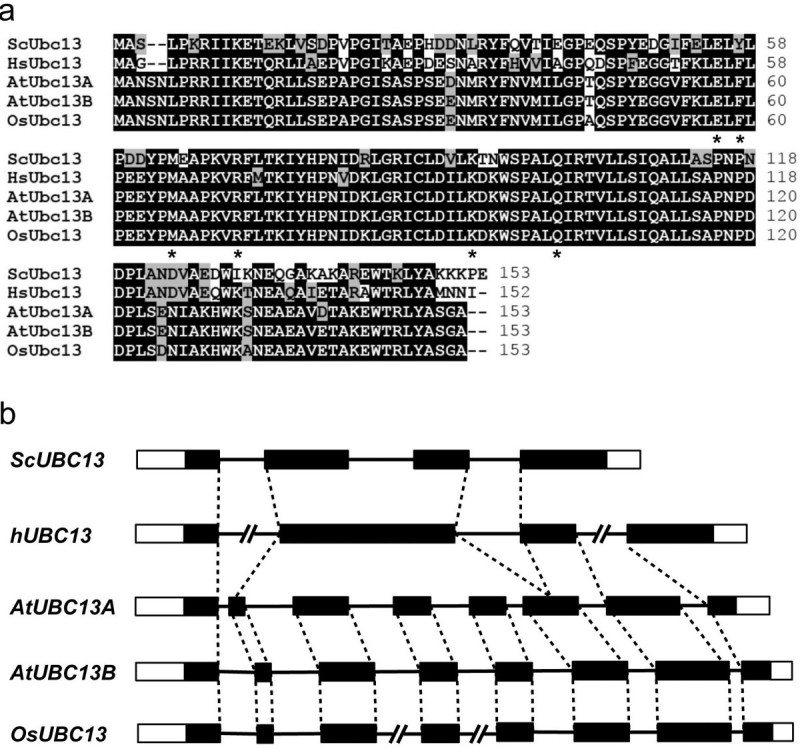
**Sequence analysis of OsUbc13 and its gene structure. a** Protein sequence alignment of OsUbc13 and Ubc13s from three other organisms. The sequences were aligned and edited using the BioEdit program version 7.0.9. Identical residues are highlighted with black while conserved residues are grey. Critical residues for Ubc13 functions are indicated with asterisks underneath the residue. **b** Genomic organization of *UBC13* genes from different organisms. Open boxes, untranslated region; filled boxes, coding regions; lines, introns; broken lines, introns longer than their proportional representation; dotted lines, identical intron-exon alignment between different *UBC13* genes. All intron-exon junctions are identical among *AtUBC13A*, *AtUBC13B* and *OsUBC13*.

To identify the rice *Ubc13* gene, a pair of highly conserved Arabidopsis *UBC13* genes was used to BLAST the rice genomic database on NCBI. Only one hypothetical gene (LOC_Os01g48280) with a high degree of sequence identity was found in the rice genome; it was named *OsUBC13*. *OsUBC13* encodes a protein of 153 amino acids with an estimated molecular weight of approximately 17 kDa. The amino acid sequence comparison of OsUbc13 with its counterparts in some other species reveals that it differs from two Arabidopsis Ubc13s, AtUbc13A and AtUbc13B, by 5 and 3 amino acids, respectively (Figure 1a). OsUbc13 also shows a high degree of conservation with Ubc13s from other eukaryotic organisms. Moreover, critical amino acid residues defined in hUbc13, including Cys87 in the active site for Ub thioester formation (McKenna et al. [2001]), Met64 for the physical interaction with RING finger E3 ligases (Wooff et al. [2004]), Glu55, Phe57 and Arg70, which form a “pocket” that determine the specific interaction with Mms2 (Pastushok et al. [2005]), as well as a Q100 residue as the target of bacterial OspI deamidation (Sanada et al. [2012]), are all conserved in OsUbc13 (Figure 1a). Genomic DNA sequence analysis as shown in Figure 1b indicate that yeast, plant and mammalian *UBC13* s are derived from the same origin in evolution, since they also share some intron-exon junctions. To further strengthen this argument, we performed phylogenetic analysis of Ubc13 and its closely related Ubcs from several model organisms ranging from yeast to plants to human. As shown in Additional file 1: Figure S1, all Ubc13s stay in the same branch away from other most closely related Ubcs, confirming that *UBC13* orthologs from entire eukaryotes are derived from the same origin. Although *AtUBC13A* and *AtUBC13B* share the same intron-exon junctions, several of their intron lengths are different. Interestingly, all except two *OsUBC13* introns are different from that of *AtUBC13B*, consistent with the amino acid alignment indicating that *OsUBC13* is more closely related to *AtUBC13B* than to *AtUBC13A*.

### *OsUBC13* Functionally complements yeast *ubc13* null mutant

**Figure 2:**
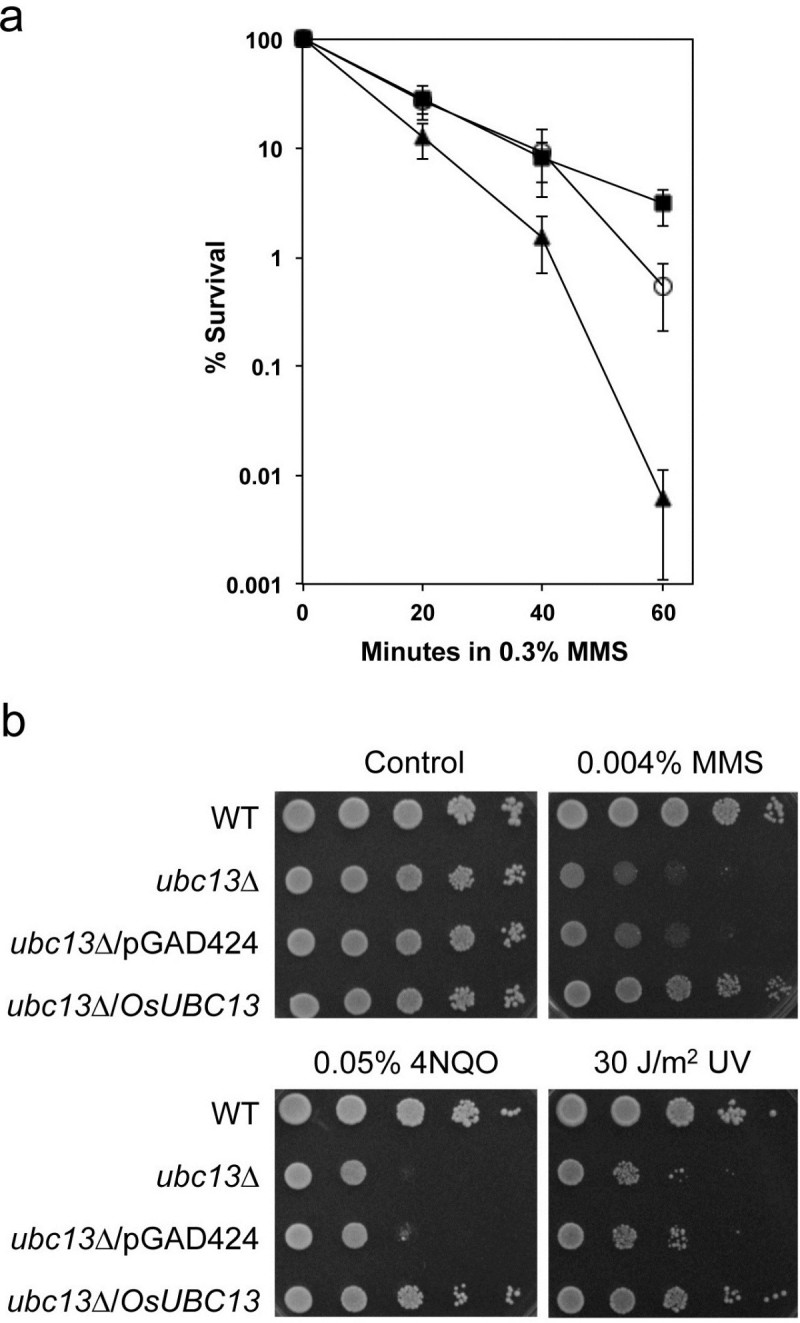
**Functional complementation of yeast ubc13 null mutant by OsUBC13. a** Protection of the *ubc13* mutant by *OsUBC13* from MMS-induced DNA damage by a liquid killing experiment. Cells were grown in`rich medium 30 min prior to the treatment. Results are from the average of at least three independent experiments with standard deviations. (■) HK578-10D (wild type); (▴) WXY904 (*ucb13*) and (○) WX904 transformed with pGAD-OsUbc13. **b** Protection of the *ubc13* mutant by *OsUBC13* from representative DNA-damaging agents by a serial dilution assay. Cells were grown overnight in SD selective media, diluted and treated as previously described (Wang et al. [2011]). Photographs were taken after 2-day incubation at 30°C.

Yeast *UBC13* plays a critical role in protecting cells from mutagenesis and cell death caused by DNA-damaging agents (Brusky et al. [2000]). In order to determine whether *OsUBC13* could functionally complement the error-free PRR defect in the yeast *ubc13* null mutant, we performed yeast killing and spontaneous mutagenesis assays. As shown in Figure 2a, expression of *OsUBC13* from a yeast two-hybrid vector rescued the *ubc13* null mutant from methyl methanesulfonate (MMS)-induced killing by up to 100-fold. It was noticed that while *OsUBC13* protects yeast *ubc13* mutant cells to a full wild-type level after 20- and 40-minute treatment, only partial complementation was achieved after 60-minute treatment. We suspect that it was due to either the nature of heterologous expression, or plasmid loss since cells were grown in the absence of selection shortly before and during MMS treatment. To further assess the *OsUBC13* function in yeast cells, a semi-quantitative serial dilution assay was performed in the presence of a few representative DNA-damaging agents. As shown in Figure 2b, *OsUBC13* rescues the yeast *ubc13* mutant from killing by MMS (alkylation damage), 4-nitroquinoline 1-oxide (4NQO, bulky lesions) and UV irradiation to a level comparable to that in wild-type cells, whereas the vector alone has no rescuing effect.

Another complementary assay was based on astonishing phenotypes of an *mms2* (Broomfield et al. [2001]) or *ubc13* (Brusky et al. [2000]) mutant with massive increase in the spontaneous mutagenesis rate, providing strong evidence that *UBC13* plays a critical role in maintaining genomic stability of host cells. Indeed, deletion of *UBC13* causes a 25-fold increase in the spontaneous mutation rate; when these *ubc13* mutant cells were transformed with a plasmid containing *OsUbc13*, the spontaneous mutation rate was reduced to about fivefold (Table 1). The residual increase in spontaneous mutagenesis above the wild type level was largely due to plasmid loss in a certain fraction of transformed cells (established to be 10-20%, data not shown) under non-selective conditions. Taken together, the above two sets of experiments allow us to conclude that *OsUbc13* functionally complements the defects of the yeast *ubc13* null mutant in the error-free PPR pathway.

**Table 1:** **Spontaneous mutation rates**

Strain^a^	Key alleles	Rate^b^(×10^-8^)	Fold increase^c^
DBY747	Wild type	1.28 ± 0.30	1
WXY849	*ubc13Δ*	31.43 ± 9.48	24.55
WXY849/OsUbc13	*ubc13Δ/OsUBC13* ^d^	6.76 ± 0.33	5.28

^a^All strains are isogenic derivatives of DBY747.

^b^The spontaneous mutation rates are the average of at least three. independent experiments with standard deviations.

^c^Relative to the wild type mutation rate.

^d^*OsUBC13* cloned in pGAD424, as used in the yeast two-hybrid assay.

### OsUbc13 Physically interacts with yeast and human E2 variants

**Figure 3:**
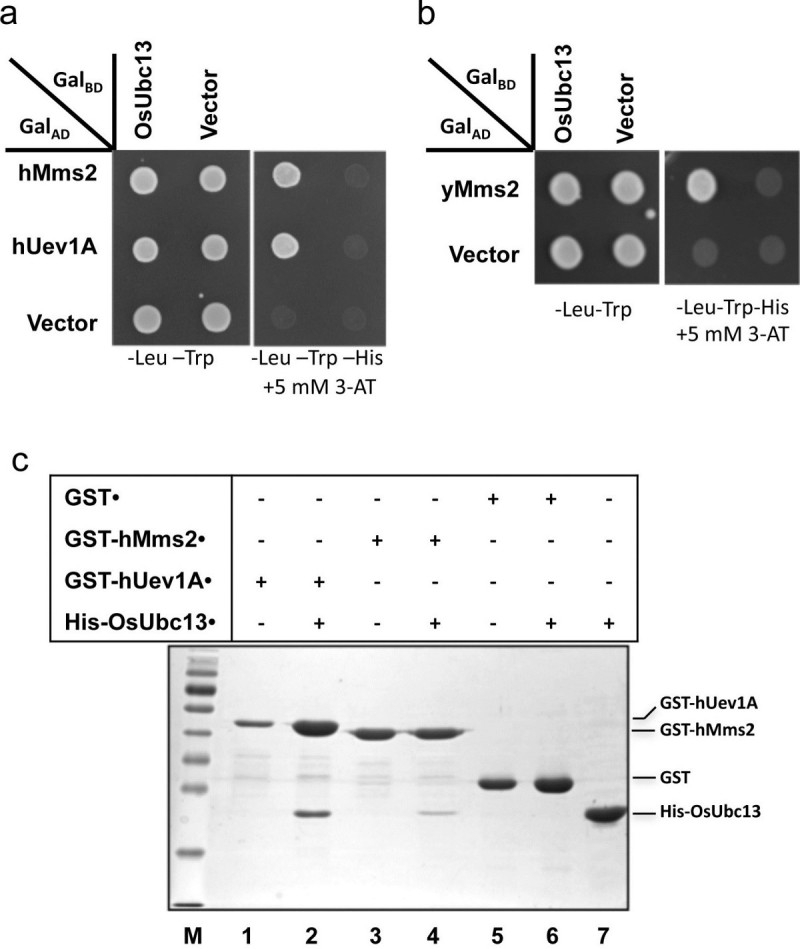
**Physical interaction of OsUbc13 with Mms2/Uev1A from other species. a** Physical interaction between OsUbc13 and human Mms2/Uev1A in a yeast two-hybrid assay. **b** Physical interaction between OsUbc13 and yeast Mms2 in a yeast two-hybrid assay. **a,b** The PJ69-4A transformants carrying one Gal4_AD_ (from pGAD424 derivative) and one Gal4_BD_ (from pGBT9 derivative) were replicated onto various plates as indicated and incubated for 3 days before being photographed. The result is representative of at least five independent transformants from each treatment. **c** Protein interactions between OsUbc13 and human Mms2/Uev1A by an affinity pull-down assay. A crude whole-cell extract from co-transformed *E. coli* cells was incubated with 100 μl GST beads for 2 hr at 4°C. The beads were then boiled with SDS-PAGE loading buffer for 5 min before SDS-PAGE analysis. GST-hUev1A (Lane 1), GST-hMms2 (Lane 3) , GST (Lane 5) and His-OsUbc13 (Lane 7) alone served as controls. pGEX6 (GST) co-transformed with His-OsUbc13 (Lane 6) also served as a negative control.

It has been known that, both in yeast and human, the assembly of K63-linked poly-Ub chains requires Ubc13 and a Uev to form a stable heterodimer ([Hofmann and Pickart 1999]; McKenna et al. [2001]; VanDemark et al. [2001]). In order to detect this interaction, a yeast two-hybrid assay ([Fields and Song 1989]) was performed between the human Uev (hUev1A and hMms2), yeast Uev (yMms2) and OsUbc13. Co-expression of Gal4_BD_-OsUbc13 with either human Uevs (Figure 3a) or yMms2 (Figure 3b) Gal4_AD_ fusion enabled cell growth in the minimal medium containing up to 5 mM 1,2,4-aminotriazole (3-AT). This is believed to be due to a complex formation that brought Gal4_AD_ and Gal4_BD_ in proximity at the *P*_*GAL1*_ promoter that resulted in the activation of a cellular *P*_*GAL1*_*-HIS3* reporter gene. All of the above interactions are robust and deemed strong, as none of the negative controls reveal positive interactions under low stringency and many bona fide positive interactions may not survive as low as 1 mM 3-AT concentration under the same experimental conditions (data not shown).

To further confirm the yeast two-hybrid results, a glutathione S-transferase (GST)-affinity pull-down assay was performed (Figure 3c). In *E. coli* cells co-transformed with plasmids expressing His_6_-OsUbc13 and GST-Uev, His_6_-OsUbc13 could be co-purified by GST-hUev1A (Lane 2) and GST-hMms2 (Lane 4) from total cell lysates. As a negative control, GST alone (Lane 6) was unable to pull-down His_6_-OsUbc13 under the same experimental conditions. Furthermore, previous yeast complementation experiments indicate that heterologous Ubc13 must physically interact with the endogenous yMms2, which is the prerequisite for the functional complementation (Pastushok et al. [2005]). Taken together, these data collectively suggest that all three Uevs from human and yeast can form stable heterodimers with OsUbc13 *in vitro* and *in vivo*.

### OsUbc13 Mediates K63-linked polyubiquitination with human Uevs *in vitro*

**Figure 4:**
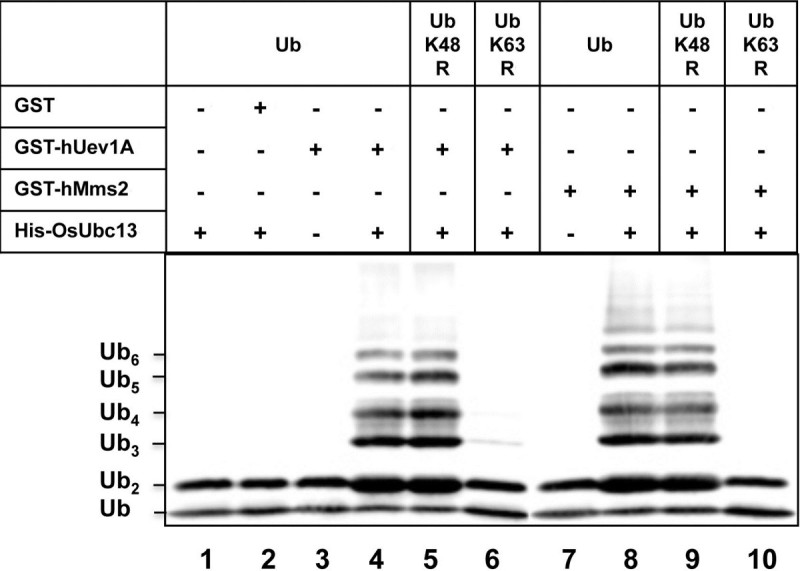
***In vitro*** **ubiquitin conjugation assays using purified proteins.** After ubiquitination reactions as described, samples were subjected to SDS-PAGE and a western blot using an anti-Ub antibody was preformed to monitor poly-Ub chain formation. The background of spontaneously formed di-Ub in the absence of E2 or Uev (Lanes 1, 3, and 7) is commonly observed in these reactions. Line 2 containing GST and OsUbc13 serves as a negative control to rule out the effect of GST tag.

Ubc13, like other Ubcs, is able to form a thioester bond with the C-terminal Ub-Gly76 at its active-site Cys residue; however, Ubc13 is the only identified E2 capable of catalyzing K63-linked polyubiquitination ([Hofmann and Pickart 1999]), and a Uev is absolutely required for this ubiquitin chain assembly. Moreover, the Ubc13-Uev complex is proved to catalyze poly-Ub chain formation through Ub-K63 instead of K48 linkage ([Hofmann and Pickart 1999]; [Hofmann and Pickart 2001]; McKenna et al. [2001]). In Arabidopsis, both AtUbc13A and AtUbc13B can promote K63-linked poly-Ub chain formation with any of the four AtUev1s (Wen et al. [2008]). In order to detect the catalytic ability of the E2 enzyme, OsUbc13, hUev1A and hMms2 were purified for an *in vitro* ubiquitination assay. As shown in Figure4, OsUbc13 (Lane 1), hUev1A (Lane 3) and hMms2 (Lane 7) alone did not promote free poly-ubiquitin chain formation. When both OsUbc13 and a Uev (hUev1A or hMms2) were present in the same reaction, di-Ub, tri-Ub and even longer Ub chains were readily formed (Lanes 4 and 8). Moreover, the assembly of free poly-Ub chains was not affected by the Ub-K48R mutation (Lanes 5 and 9); however, the Ub-K63R mutation completely abolished the Ub chain formation (Lanes 6 and 10). The above results indicate that with the assistance of hUev1A or hMms2, OsUbc13, can catalyze free poly-ubiquitin chain formation through K63 linkage.

### *OsUbc13* Is ubiquitously expressed and does not fluctuate under biotic or abiotic stresses

**Figure 5:**
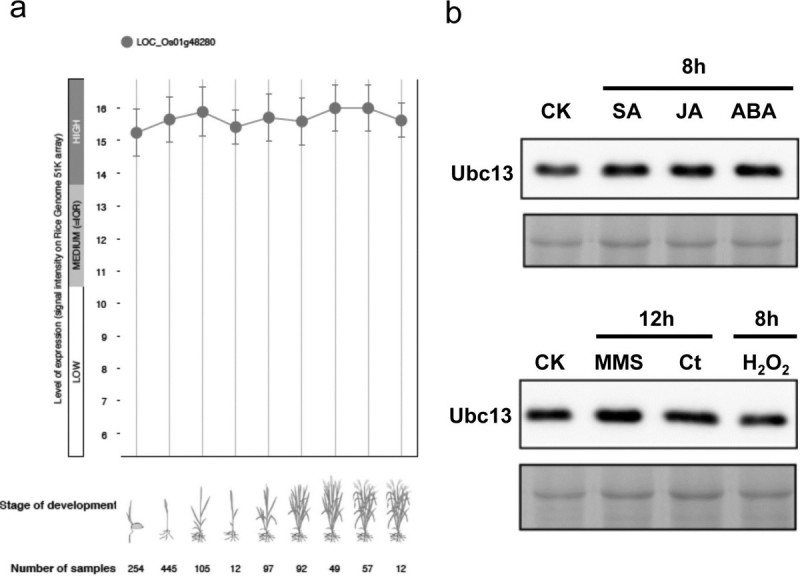
**Quantitative analyses of** ***OsUBC13*** **expression. a** Expression of *OsUBC13* (LOC_Os01g48280) during rice development. Samples were taken from different developmental stages as indicated and relative transcript levels of the entire transcriptome were determined by microarray analysis. The data is retrieved from Genevestigator (www.genevestigator.com). **b** Relative OsUbc13 protein level in rice seedlings after treatment with various stresses, two weeks after germination rice seedlings were treated with the given biotic or abiotic agent for the predetermined time as indicated on top of each panel and total proteins were extracted from the whole plants. The cellular OsUbc13 was detected by western bolt analysis using the anti-hUbc13 mAb 4E11 (Andersen et al. [2005]). Each lane contains 20 μg total protein and the most abundant protein band as visualized by Coomassie brilliant blue staining serves as a loading control (the bottom image of each panel). CK, untreated seedlings; Ct, cisplatin.

Since Ubc13 is mainly involved in stress responses such as DNA damage and innate immunity, it is reasonable to assume that *UBC13* expression may be regulated in response to these stresses. Indeed, *S. cerevisiae UBC13* is induced when cells are treated with different DNA-damaging agents (Brusky et al. [2000]). In contrast, the expression of neither Arabidopsis *UBC13* gene appears to fluctuate in different tissues or be altered by biotic or abiotic stresses (Wen et al. [2006]). To further investigate the expression profiles of *OsUBC13*, we performed a cross-database search on the Genevestigator (Hruz et al. [2008]). The retrieved data show that the expression of *OsUBC13* (LOC_Os01g48280) remains high and remarkably stable during development (Figure 5a) and in different tissues ( Additional file 2: Figure S2). The differences, if any, fall in experimental variation and are statistically insignificant. Moreover, none of the biotic or abiotic stress treatment induces or suppresses *OsUBC13* expression beyond twofold, and the vast majority are even less than 1.2-fold or perhaps within experimental variation ( Additional file 3: Figure S3). Hence, the expression pattern of *OsUBC13* appears to be comparable to that of *AtUBC13* genes.

Although the transcript level of *OsUBC13* is relatively stable, it does not rule out the possibility that *OsUBC13* may be regulated at the protein level. To further determine the cellular Ubc13 protein level under various stresses, we used the monoclonal antibody 4E11 produced in-house against hUbc13 (Andersen et al. [2005]), which shares a very high degree of amino acid sequence identity with OsUbc13 (Figure 1a). A single band of approximately 20 kDa was detected from total rice seedling extract in a western blot analysis, consistent with the predicted OsUbc13 protein size. In this study, abscisic acid (ABA), salicylic acid (SA) and jasmonic acid (JA) were selected to represent hormone treatments, while methyl methanesulfonate (MMS), cisplatin and hydrogen peroxide (H_2_O_2_) were chosen to represent DNA damage treatments. As shown in Figure 5b and quantitated by densitometry (data not shown), no obvious alteration was observed by any given treatments under our experimental conditions.

## Discussion

While sunlight is absolutely necessary for photosynthesis by land plants, the threats of solar UV radiation as well as other genotoxic chemicals are also critical to plants survival due to their sessile living style. As the major food resources for human beings, crops may severely lose yield and quality due to the above stresses, which compromise global food security. In this study, we isolated and characterized the rice *UBC13* gene, which is believed to be the first reported crop gene involved in the DNA damage-tolerance pathway. Our *in vitro* studies confirmed that the highly conserved OsUbc13 protein could physically interact with the cognate E2 variants from yeast and human. Functional examination revealed that *OsUBC13* could restore cellular activities of the yeast *ubc13* null mutant to limit spontaneous mutagenesis and provide resistance to DNA-damaging agents. Since yeast *UBC13* plays an essential role in mediating error-free DDT (Broomfield et al. [1998]), these results suggest that *OsUBC13* is involved in the error-free DDT pathway in rice as well, although definitive evidence has to wait for the characterization of *UBC13* knockdown or knockout rice plants. It is noticed that rice may be a more suitable experimental plant model than other model plants including Arabidopsis, since it contains only one predicted *UBC13* gene, while Arabidopsis contains two highly conserved and most likely redundant *UBC13* genes (Wen et al. [2006]). Furthermore, since the DDT pathway appears to be conserved from yeast to humans (Andersen et al. [2005]), this study may serve as a gateway to understanding DDT mechanisms in rice in an attempt to enhance its resistance to this major environment stress.

Ubc13 is the only known E2 that catalyzes K63-linked polyubiquitination. This catalytic activity is dependent on its interaction with an E2 variant (Uev) and Ubc13 associated with different Uevs is thought to mediate different cellular processes (Andersen et al. [2005]). In *S. cerevisiae*, Ubc13 has only one Uev partner, Mms2, and the Ubc13-Mms2 heterodimer is required for the K63 polyubiquitination of PCNA (Hoege et al. [2002]). In human cells, two Ubc13-associated Uevs are found (Xiao et al. [1998]), and they appear to play different roles (Andersen et al. [2005]): the Ubc13-hMms2 complex is required for DNA-damage response, while the Ubc13-Uev1A complex is required for the NF-κB signaling pathway (Deng et al. [2000]) through activation of NEMO (Zhou et al. [2004]) and/or TAK1 (Wang et al. [2001]). In this study, we found that OsUbc13 forms stable complexes with both hMms2 and Uev1A, and could catalyze K63-linked poly-Ub chains *in vitro* with either Uev1A or hMms2. Interestingly, Arabidopsis contains four *UEV1* genes; at least one of them (*UEV1D*) is involved in DNA-damage response, while at least one (*UEV1A*) is not required for this response (Wen et al. [2008]). Since the rice genome also contains multiple *UEV1* homologs (Y.Z. and W.X., data not shown), it is conceivable that rice Ubc13 associates with different Uev1s to catalyze K63-linked poly-Ub chains. These different heterodimers may interact with different E3s, target proteins and hence are involved in various stress responses including, but not limited to, DNA-damage tolerance. Indeed, Ubc13-mediated ubiquitination has been reported to be involved in cellular processes such as neurodegeneration (Doss-Pepe et al. [2005]; Lim et al. [2005]; [Muralidhar and Thomas 1993]; Oh et al. [1994]), homologous recombination (Doil et al. [2009]; Huen et al. [2007]; Mailand et al. [2007]; Stewart et al. [2009]), innate immunity (Deng et al. [2000]; Zhou et al. [2004]), cell cycle checkpoint (Bothos et al. [2003]; Laine et al. [2006]; Wen et al. [2012]), as well as development (Yin et al. [2007]) and iron metabolism ([Li and Schmidt 2010]) in Arabidopsis. Some of these processes may not necessarily require a Uev (Huen et al. [2008]) or involve K63-linked polyubiquitination.

K63-linked polyubiquitination process is primarily involved in stress responses, within which the E2 complex plays a critical role. It is reasonable to assume that the Ubc13-Uev activity is tightly regulated in response to stress. In *S. cerevisiae*, *UBC13* is transcriptionally regulated in response to DNA damage. In higher eukaryotes, because Ubc13 is involved in multiple cellular processes, and whose specificity is largely determined by the cognate Uev, regulation of Uev activity would be evolutionarily preferred. Indeed, there is little experimental evidence on altered Ubc13 activity in mammalian cells, while cellular Uev levels appear to fluctuate in different tissues and in response to environmental stresses (Fritsche et al. [1997]; Ma et al. [1998]; Sancho et al. [1998]). Of great interest is the observation that transcript levels of *UBC13* genes in Arabidopsis and rice are relatively stable regardless of plant development, tissue distribution and response to stresses, indicating that *UBC13* is a housekeeping gene in plants. In sharp contrast, the expression of plant *UEV1* genes fluctuate under the same experimental conditions, suggesting that plants achieve the control of K63 polyubiquitination through differential regulation of *UEV1* genes, or that Uevs serve as a regulatory subunit for K63 ubiquitination. This molecular evolution further testifies that in higher eukaryotes, K63 ubiquitination is involved in multiple cellular processes.

Since transcriptional regulation is only one of several means of controlling target gene activity, in this study we attempted to assess the cellular Ubc13 protein level under different stress conditions. Western blot analysis by using an anti-hUbc13 monoclonal antibody further confirms that *UBC13* is a candidate housekeeping gene. Hence, we recommend Ubc13 to be a suitable candidate to serve as an internal reference for rice-related research because it fulfills the following criteria: (a) it is a highly expressed gene; (b) its expression level is remarkably constant under various growth stages, tissues and experimental conditions; (c) there is only one copy of the homologous gene; and (d) the monoclonal antibody 4E11 used in this study is commercially available.

## Conclusions

The present study describes the isolation and functional characterization of the rice *UBC13* (*OsUBC13*) gene and its product. Our observations collectively suggest that Ubc13-mediated K63 polyubiquitination is conserved in rice, and that *OsUBC13* may be involved in DNA damage tolerance as well as other cellular stress responses. Furthermore, bioinformatic analysis and our experimental data suggest that *OsUBC13* is a housekeeping gene, and that its expression can be utilized as an internal control for other rice-related investigations.

## Methods

### Plant materials and yeast cell culture

Rice (*Oryza sativa L. cv. Japonaca*) seeds were surface sterilized with 2% NaClO for 30 min after pre-wash by sterilized distilled water, followed by washing seven times in sterilized water. The sterilized rice seeds were plated in ½ Murashige and Skoog (MS) plates containing MS with 2.2 g/l minimal organics (Sigma M6899), 10 g/l sucrose and 1% agar (BD 214010), and vertically cultured in a growth chamber (16 h light/8 h dark and 30°C constant). Two weeks after seed germination, rice seedlings were transferred to ½ MS solution for an additional 1-day incubation. For ABA (final concentration 50 μM), MMS (final concentration of 0.175%) and cisplatin (final concentration 16 μM) treatments, stock solutions were added to the nutrient solution to the predetermined final concentration. For SA and meJA treatments, SA (5 mM in water) and meJA (0.1 mM in 0.1% ethanol) were sprayed onto the rice shoot. For ABA, SA, meJA and H_2_O_2_ treatments, the seedlings were harvested after 8 hours. For MMS and cisplatin treatments, seedlings were harvested after 12 hours.

The haploid yeast strains used in this study are listed in Additional file 4: Table S1. Yeast cells were grown at 30°C in either rich YPD or in a synthetic dextrose (SD) medium (0.67% Bacto-yeast nitrogen base without amino acids, 2% glucose) supplemented with necessary nutrients as recommended (Sherman et al. [1983]). For solid plates, 2% agar was added to either YPD or SD medium prior to autoclaving. Yeast cells were transformed using a LiAc method as described (Ito et al. [1983]). The source and preparation of *ubc13Δ::HIS3* cassettes was as previously described (Xiao et al. [2000]).

### Cloning rice *UBC13* cDNA and plasmid construction

To clone the full-length open reading frame (ORF) of *OsUBC13*, total RNA was isolated from rice seedlings by using TRIzol reagents (Invitrogen, Carlsbad, CA, USA) for RT-PCR with the RevertAid First Strand cDNA Synthesis Kit (Fermentas) following the manufacturer’s protocols. *OsUbc13* specific primers are as follow: 5’-aatccggaattc ATGGCCAACAGCAACCTC-3’ and 5’-atacgcgtcgac TTATGCACCGCTGGC-3’. The forward primer contains an *Eco* RI restriction site and the reverse primer contains a *Sal* I site, as underlined. The PCR product of *OsUBC13* ORF was cloned into yeast two-hybrid vectors pGBT9Bg and pGAD424Bg, which were derived from pGBT9 and pGAD424 ([Bartel and Fields 1995]), respectively.

### Yeast two-hybrid analysis

The yeast two-hybrid strain PJ69-4A (James et al. [1996]) was used for this assay. The co-transformation, selection and two-hybrid detection steps were as previously described (Wen et al. [2006]).

### Recombinant protein purification and ubiquitination assay

The *OsUbc13* ORF was isolated from pGBT-OsUbc13 and cloned into pET-30a. The resulting pET-OsUbc13 was transformed into BL21 CodonPlus (DE3)-RIL cells. The His_6_-OsUbc13 fusion protein was purified following a previously published protocol (Anderson et al. [2008]). Meanwhile, GST, GST-hUev1A and GST-hMms2 were produced and purified as previously described (Andersen et al. [2005]). For an *in vitro* ubiquitination assay, the 20-μl reaction mixture contained 225 nM E1 enzyme, 450 μM Ub, 1 mM Mg-ATP, 1 μM Ubc13, and 1 μM Uev1A in the supplied reaction buffer (Boston Biochem). The K63R and K48R mutant Ub proteins were also purchased from Boston Biochem. The conjugation reactions were performed at 37°C for 2 hr. Samples were subjected to SDS-PAGE (12%), and Ub and poly-Ub were detected through protein gel blots using polyclonal rabbit anti-Ub antibodies (Sigma-Aldrich).

### GST pull-down assay

The BL21 competent cells were transformed with either pGEX6p-1, pGEX-hMms2 or pGEX-hUev1A alone, or co-transformed with pET-OsUbc13. The whole cell extracts were incubated with Glutathione Sepharose 4B Microspin^TM^ beads in 4°C for 2 hr. The beads were collected by centrifugation and washed with a lysis buffer 5 times. Finally, beads were boiled with loading buffer and analyzed on a 12% SDS-PAGE gel.

### Rice protein extraction and western blot analysis

Two-week-old rice seedlings were homogenized in liquid nitrogen. Total protein was extracted using a buffer containing 50 mM Tris–HCl pH8.0, 0.3 M NaCl, 2 mM EDTA, 10% Glycerol, 0.1% TritonX-100, 10 mM PMSF, 3 mM DDT and 1/5 tablet per 10 ml protease inhibitor (Roche). The extract was centrifuged at 16,000 *g* for 10 min at 4°C. The supernatant was then transferred to a new tube and centrifuged twice prior to SDS/PAGE analysis. The OsUbc13 protein in each sample was detected by western bolt using the anti-hUbc13 monoclonal antibody 4E11 (Andersen et al. [2005]).

### Yeast cell survival assays

Yeast strain HK578-10D and its isogenic *ubc13Δ* single mutant transformed with pGAD424 or pGAD-OsUbc13, were used in this assay. The yeast liquid killing and serial dilution assays were performed as previously described (Barbour et al. [2006]; Wang et al. [2011]). For the serial dilution assay, several doses for each DNA-damaging agent were employed and only one representative dose is presented.

### Spontaneous mutagenesis assay

Yeast strain DBY747 and its isogenic *ubc13Δ* derivative WXY849 bear a *trp1-289* amber mutation that can be reverted to Trp^+^ by several different mutation events ([Xiao and Samson 1993]). WXY849 was transformed with pGAD-OsUbc13 and the spontaneous mutagenesis assay was performed as described previously (Anderson et al. [2008]).

## Electronic supplementary material

Additional file 1:**Figure S1.** Phylogenetic analysis of selected ubiquitin conjugating enzyme (E2) family proteins based on the alignment of 59 protein sequences from 7 species (*S. cerevisiae, A. thaliana, O. sativa, D. melanogaster, C. elegans, H. sapiens, D. rerio* **).** The circled clade represents Ubc13s derived from NP_010377.1 (*S. cerevisiae*), NP_564011.1 and NP_849902.2 (*A. thaliana*), NP_001043834.1 (*O. sativa*), NP_001162752.1 (*D. melanogaster*), NP_500272.2 (*C. elegans*), NP_003339.1 (*H. sapiens*), NP_998651.1 (*D. rerio*). Note that the mouse and human Ubc13 sequences are identical (data not shown). Five other human Ubcs (UBE2D, UBE2E, UBE2J, UBE2K and UBE2T) that most closely related to Ubc13 in sequence and their orthologs in the above species were retrived for sequence alignment. The phylogenetic tree was drawn by using a MEGA 5.05 program. (PDF 275 KB)

Additional file 2:**Figure S2.** Quantitative analysis of *OsUBC13* (LOC_Os01g48280) expression in rice tissues. Samples were taken from different rice tissues as indicated in the left column and relative transcript levels in each tissue-specific transcriptome were determined by microarray analysis. The data is retrieved from Genevestigator (http://www.genevestigator.com). (PDF 59 KB)

Additional file 3:**Figure S3.** Quantitative analysis of *OsUBC13* (LOC_Os01g48280) expression in rice under different stress conditions. Samples were taken from rice tissues after the plants were treated as indicated in the left column. Relative expression levels were determined by microarray analysis. The resulting data were compiled and retrieved from Genevestigator (http://www.genevestigator.com), and displayed in log2 scale in the middle column. The actual fold change values upon stress treatment are given in the right column. (PDF 215 KB)

Additional file 4:**Table S1.** *Saccharomyces cerevisiae* strains. (DOCX 51 KB)

Below are the links to the authors’ original submitted files for images.

Authors’ original file for figure 1

Authors’ original file for figure 2

Authors’ original file for figure 3

Authors’ original file for figure 4

Authors’ original file for figure 5
