# Supplementary figures and images for: Rice UBC13, a candidate housekeeping gene, is required for K63-linked polyubiquitination and tolerance to DNA damage
Source: Rice (N Y). 2012 Sep 8;5:24. doi: 10.1186/1939-8433-5-24 (PMC5520843; doi:10.1186/1939-8433-5-24)

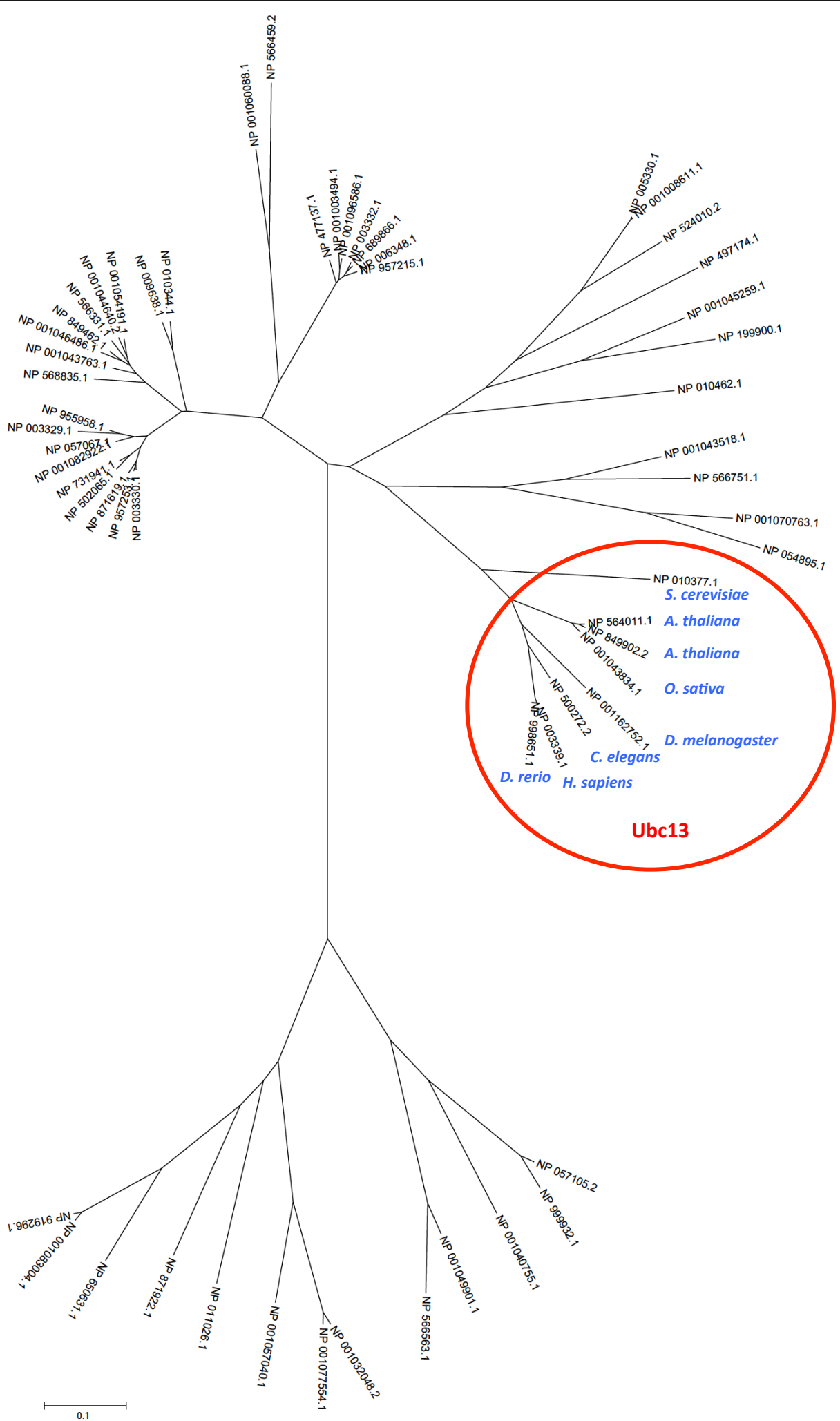

Supplement: Supplementary file 1 — Additional file 1:Figure S1. Phylogenetic analysis of selected ubiquitin conjugating enzyme (E2) family proteins based on the alignment of 59 protein sequences from 7 species (S. cerevisiae, A. thaliana, O. sativa, D. melanogaster, C. elegans, H. sapiens, D. rerio ). The circled clade represents Ubc13s derived from NP_010377.1 (S. cerevisiae), NP_564011.1 and NP_849902.2 (A. thaliana), NP_001043834.1 (O. sativa), NP_001162752.1 (D. melanogaster), NP_500272.2 (C. elegans), NP_003339.1 (H. sapiens), NP_998651.1 (D. rerio). Note that the mouse and human Ubc13 sequences are identical (data not shown). Five other human Ubcs (UBE2D, UBE2E, UBE2J, UBE2K and UBE2T) that most closely related to Ubc13 in sequence and their orthologs in the above species were retrived for sequence alignment. The phylogenetic tree was drawn by using a MEGA 5.05 program. (PDF 275 KB) [file 12284_2012_18_MOESM1_ESM.pdf]

● LOC\_Os01g48280

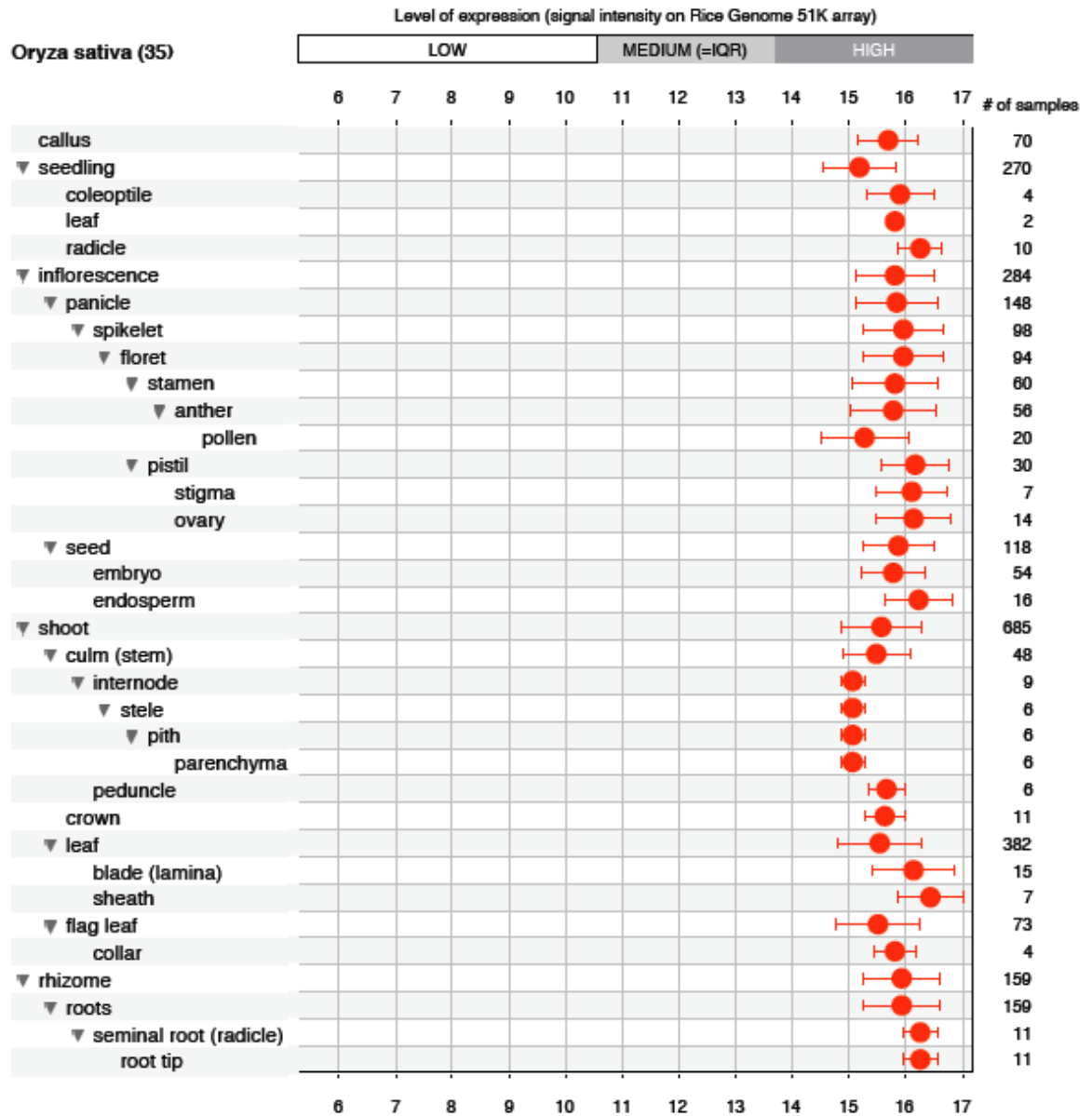

Figure S2

Supplement: Supplementary file 2 — Additional file 2:Figure S2. Quantitative analysis of OsUBC13 (LOC_Os01g48280) expression in rice tissues. Samples were taken from different rice tissues as indicated in the left column and relative transcript levels in each tissue-specific transcriptome were determined by microarray analysis. The data is retrieved from Genevestigator (http://www.genevestigator.com). (PDF 59 KB) [file 12284_2012_18_MOESM2_ESM.pdf]

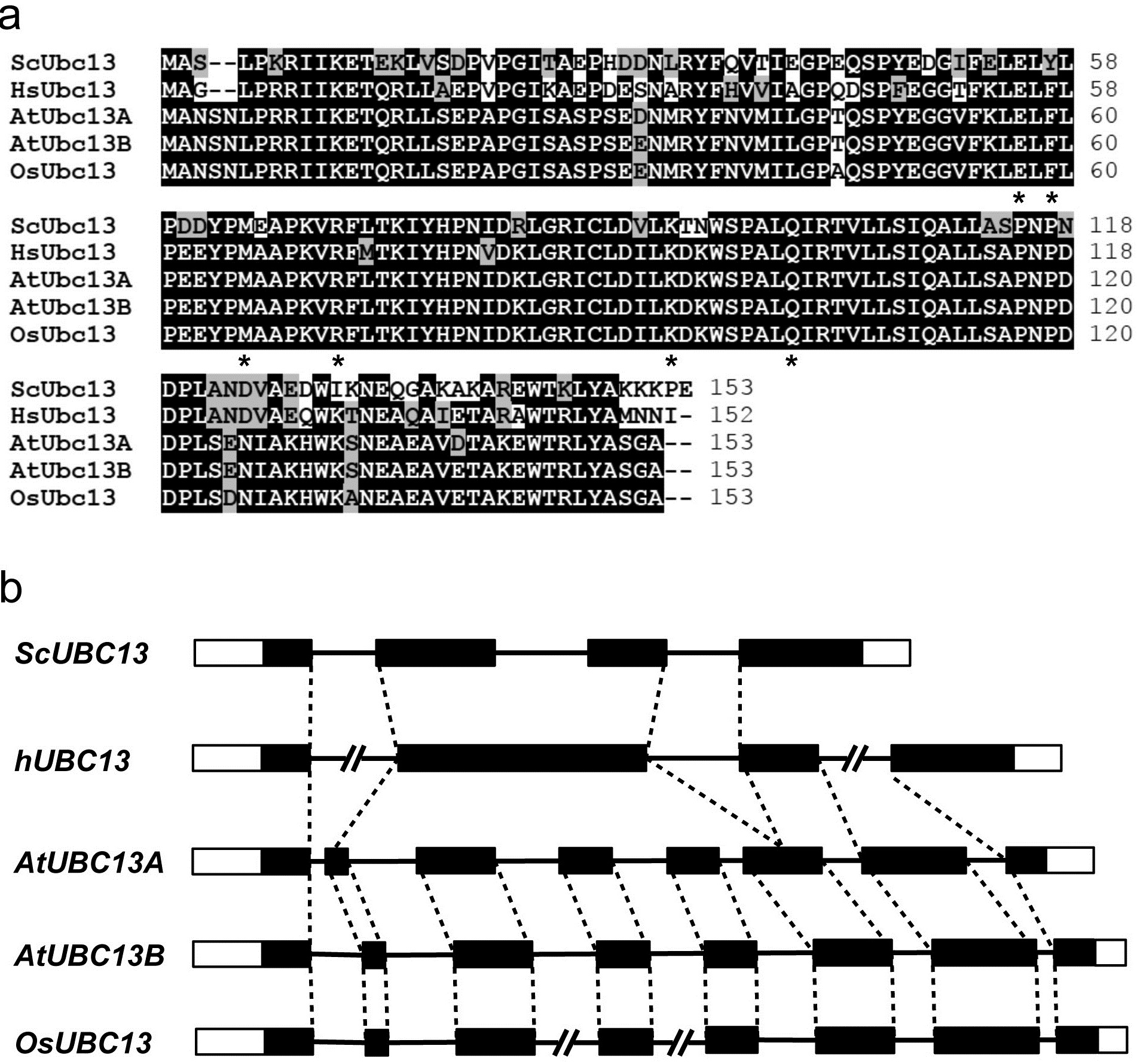

Supplement: Supplementary file 5 — Authors’ original file for figure 1 [file 12284_2012_18_MOESM5_ESM.jpeg]

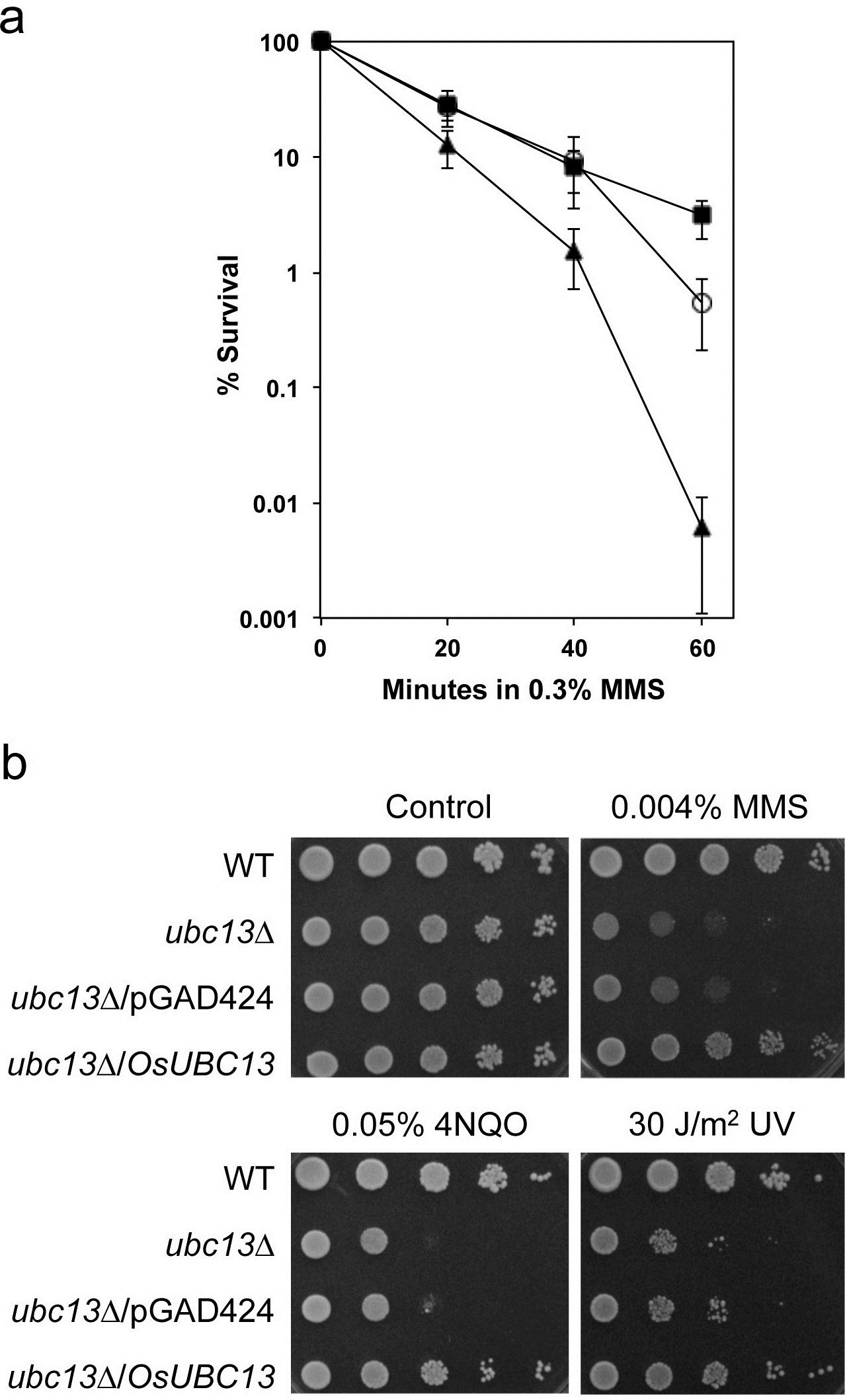

Supplement: Supplementary file 6 — Authors’ original file for figure 2 [file 12284_2012_18_MOESM6_ESM.jpeg]

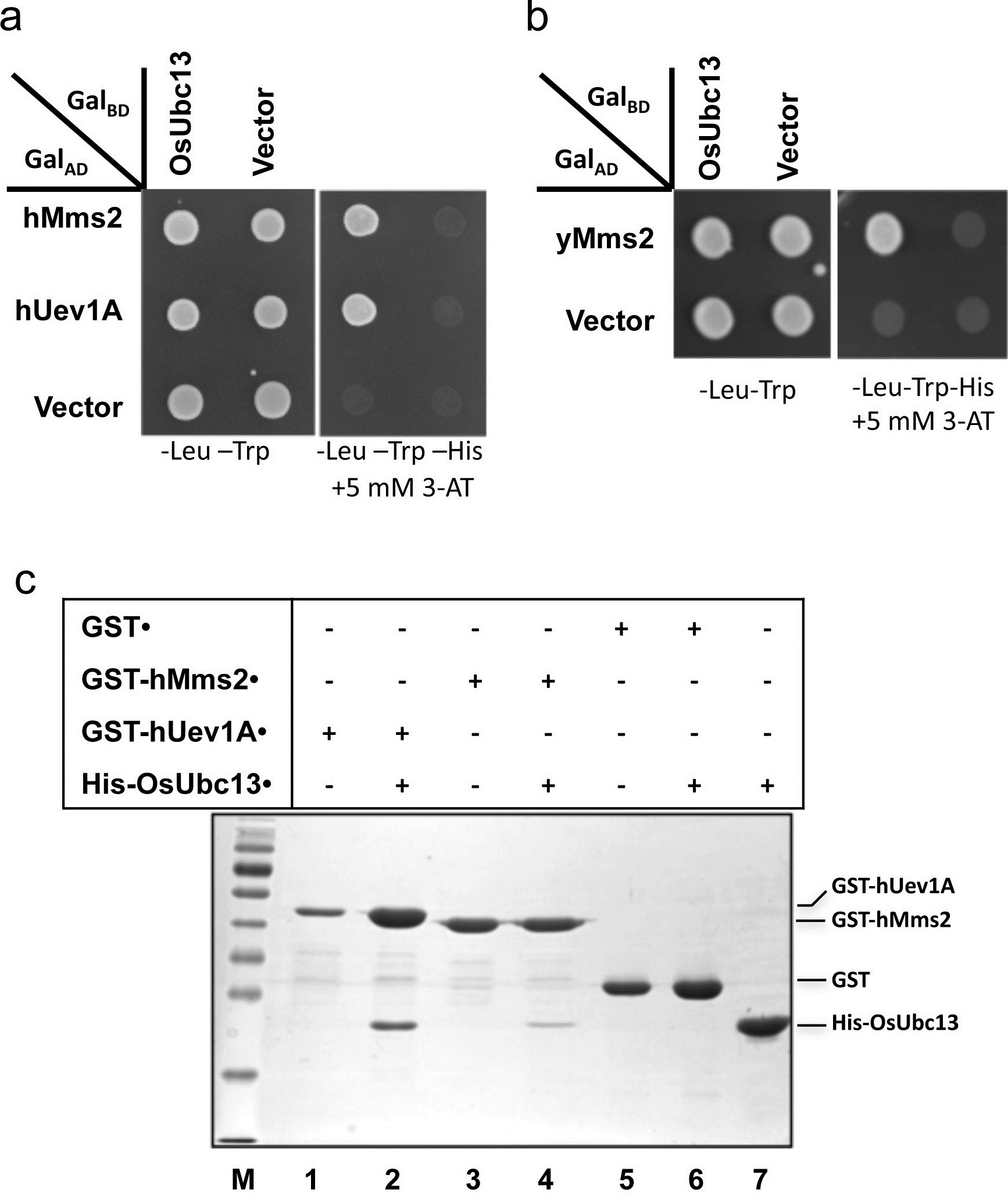

Supplement: Supplementary file 7 — Authors’ original file for figure 3 [file 12284_2012_18_MOESM7_ESM.jpeg]

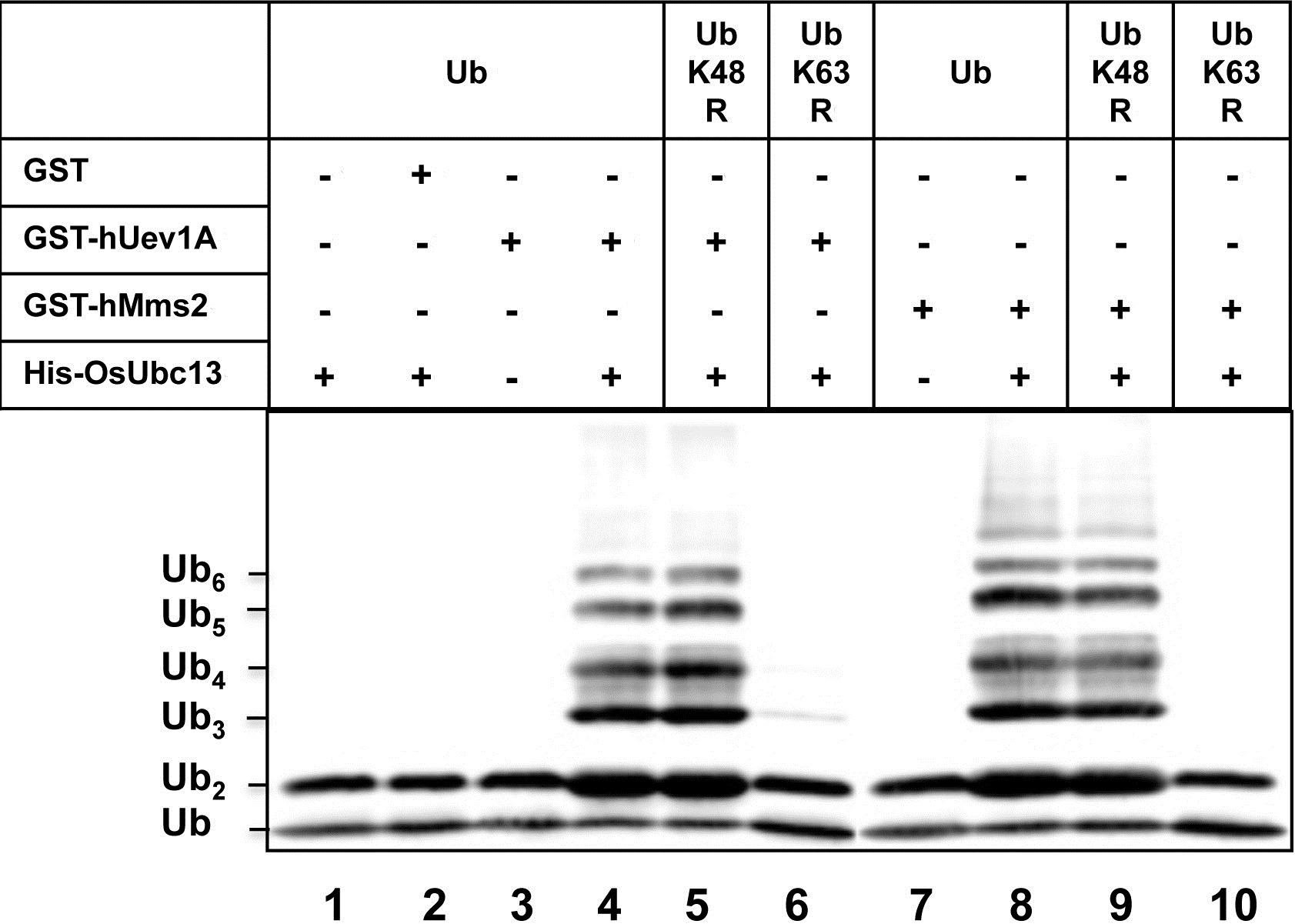

Supplement: Supplementary file 8 — Authors’ original file for figure 4 [file 12284_2012_18_MOESM8_ESM.jpeg]

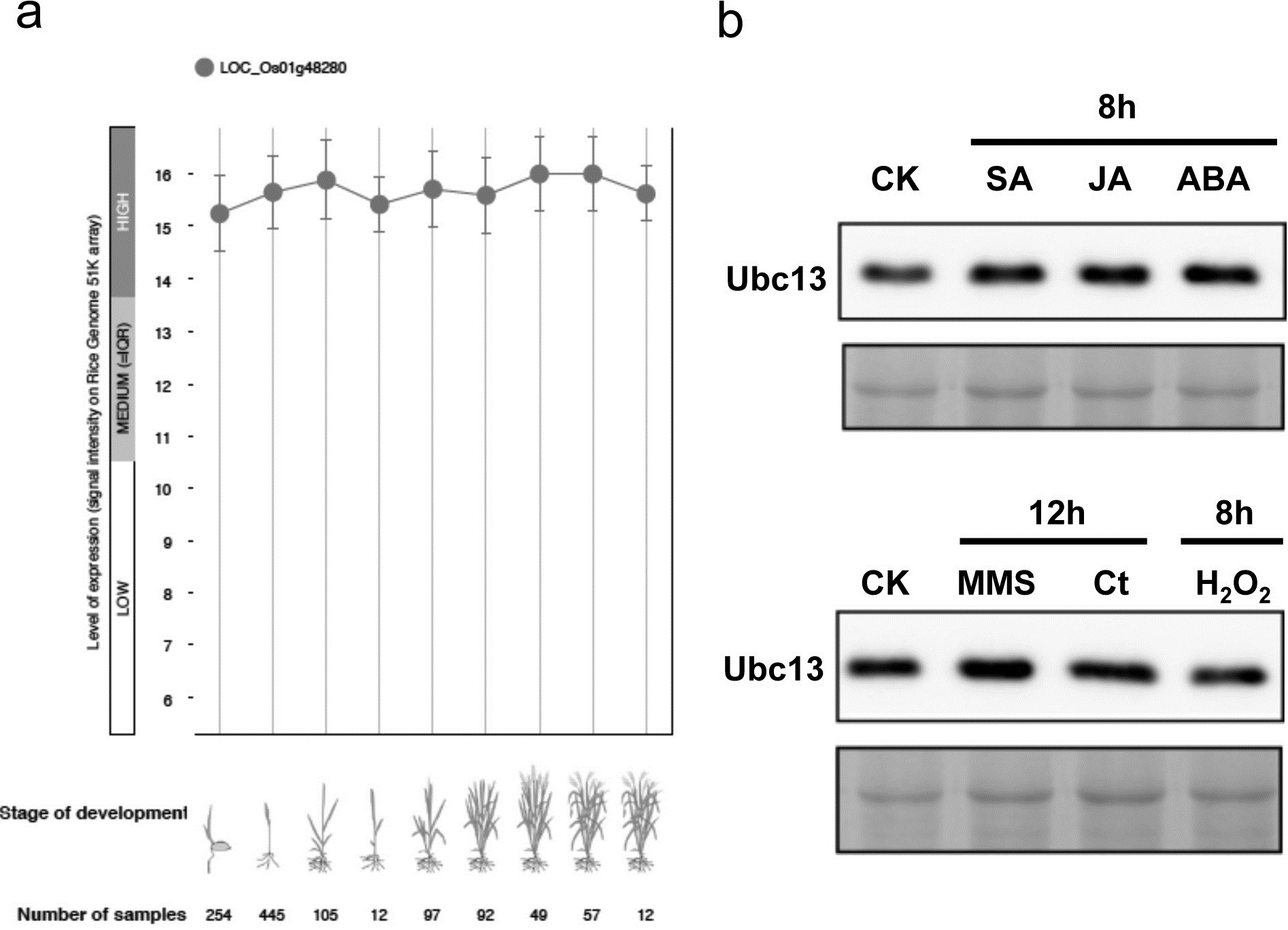

Supplement: Supplementary file 9 — Authors’ original file for figure 5 [file 12284_2012_18_MOESM9_ESM.jpeg]
