# Supplementary material for: Rice UBC13, a candidate housekeeping gene, is required for K63-linked polyubiquitination and tolerance to DNA damage
Source: Rice (N Y). 2012 Sep 8;5:24. doi: 10.1186/1939-8433-5-24 (PMC5520843; doi:10.1186/1939-8433-5-24)
Supplement: Supplementary file 3 — Additional file 3:Figure S3. Quantitative analysis of OsUBC13 (LOC_Os01g48280) expression in rice under different stress conditions. Samples were taken from rice tissues after the plants were treated as indicated in the left column. Relative expression levels were determined by microarray analysis. The resulting data were compiled and retrieved from Genevestigator (http://www.genevestigator.com), and displayed in log2 scale in the middle column. The actual fold change values upon stress treatment are given in the right column. (PDF 215 KB) [file 12284_2012_18_MOESM3_ESM.pdf]

LOC_Os01g48280

555 of 555 perturbations fulfilled the filter criteria

Filter values for LOC_Os01g48280

Oryza sativa (589)

<< down-regulated — Log(2)-ratio — up-regulated >>

< -4 | -3 | -2 | -1 | 0 | 1 | 2 | 3 | 4 >

Oryza sativa (589)	Log(2)-ratio	Fold-Change (no filter)	p-value (no filter)
▼ Biotic			
A. tumefaciens (1h) / untreated calli samples	0.08	1.05	0.573
A. tumefaciens (6h) / untreated calli samples	0.50	1.41	0.037
A. tumefaciens (Nipponbare) / untreated calli samples (Nipponbare)	0.10	1.08	0.503
A. tumefaciens (Zhenshan 97) / untreated calli samples (Zhenshan 97)	0.34	1.27	0.132
A. tumefaciens study 2 (Nipponbare) / untreated calli samples (Nipponbare)	0.59	1.55	0.094
A. tumefaciens study 2 (Zhenshan 97) / untreated calli samples (Zhenshan 97)	0.80	1.77	0.030
A. tumefaciens study 3 (Nipponbare) / untreated calli samples (Nipponbare)	0.01	1.00	0.909
A. tumefaciens study 3 (Zhenshan 97) / untreated calli samples (Zhenshan 97)	0.03	1.02	0.784
A. tumefaciens study 4 (Nipponbare) / untreated calli samples (Nipponbare)	-0.02	-1.02	0.826
A. tumefaciens study 4 (Zhenshan 97) / untreated calli samples (Zhenshan 97)	-0.09	-1.07	0.531
M. graminicola (2dpi) / untreated root samples	-0.08	-1.06	0.063
M. graminicola (4dpi) / untreated root samples	-0.12	-1.08	0.365
M. grisea (3dpi) / mock treated leaf samples (3dpi)	0.19	1.14	0.039
M. grisea (4dpi) / mock treated leaf samples (4dpi)	0.55	1.47	0.031
M. grisea study 2 (Taipei 309) / mock treated leaf samples (Taipei 309)	-0.39	-1.31	0.079
M. grisea study 2 (TP-Pi54-15) / mock treated leaf samples (TP-Pi54-15)	-0.35	-1.27	0.081
M. incognita (6dpi) / uninfected root samples	-0.23	-1.17	0.010
M. oryzae (2dpi) / mock treated root samples (2dpi)	0.04	1.03	0.496
M. oryzae (4dpi) / mock treated root samples (4dpi)	-0.02	-1.02	0.508
M. oryzae (6dpi) / mock treated root samples (6dpi)	0.08	1.06	0.181
N. lugens (Rathu Heenati) / untreated stem samples (Rathu Heenati)	-0.03	-1.02	0.638
N. lugens (Taichung Native-1) / untreated stem samples (Taichung Native-1)	0.04	1.03	0.708
N. lugens study 2 (Rathu Heenati) / untreated stem samples (Rathu Heenati)	-0.15	-1.11	0.025
N. lugens study 2 (Taichung Native-1) / untreated stem samples (Taichung Nati...	-0.26	-1.19	0.037
O. oryzae (GMB1) / untreated stem samples	0.24	1.16	0.288
O. oryzae (GMB4M) / untreated stem samples	0.21	1.14	0.456
RSV (KT95-418) / non-infected aeral tissue samples (KT95-418)	-0.15	-1.12	0.450
RSV (WuYun3) / non-infected aeral tissue samples (WuYun3)	0.02	1.01	0.788
S. hermonthica (IAC 165) / mock treated root samples (IAC 165)	0.14	1.11	0.309
S. hermonthica (Nipponbare) / mock treated root samples (Nipponbare)	0.05	1.03	0.382
S. hermonthica study 2 (IAC 165) / mock treated root samples (IAC 165)	0.06	1.04	0.380
S. hermonthica study 2 (Nipponbare) / mock treated root samples (Nipponbare)	-0.03	-1.02	0.394
S. hermonthica study 3 (IAC 165) / mock treated root samples (IAC 165)	-0.01	-1.01	0.785
S. hermonthica study 3 (Nipponbare) / mock treated root samples (Nipponbare)	-0.11	-1.08	0.293
X. campestris pv. vesicatoria (Huahui 1) / untreated leaf samples (Huahui 1)	-0.05	-1.03	0.328
X. campestris pv. vesicatoria (MH63) / untreated leaf samples (MH63)	0.10	1.07	0.211
X. oryzae pv. oryzae (24hpi) / mock treated shoot samples (24hpi)	0.02	1.01	0.799
X. oryzae pv. oryzae (2hpi) / mock treated shoot samples (2hpi)	0.11	1.08	0.385
X. oryzae pv. oryzae (4hpi) / mock treated shoot samples (4hpi)	0.09	1.07	0.321
X. oryzae pv. oryzae (8hpi) / mock treated shoot samples (8hpi)	0.04	1.03	0.812
X. oryzae pv. oryzae (96hpi) / mock treated shoot samples (96hpi)	0.03	1.02	0.865
X. oryzae pv. oryzae JXOI (Huahui 1) / untreated leaf samples (Huahui 1)	-0.13	-1.09	0.013
X. oryzae pv. oryzae JXOI (MH63) / untreated leaf samples (MH63)	0.13	1.09	0.115
X. oryzae pv. oryzae PXO86 (IR24) / mock treated leaf samples (IR24)	0.35	1.28	0.008
X. oryzae pv. oryzae PXO86 (IR24) / X. oryzae pv. oryzae T7174 (IR24	0.24	1.18	0.090
X. oryzae pv. oryzae PXO86 (IR24) / X. oryzae pv. oryzae PXO99A (IR24	0.15	1.10	0.252
X. oryzae pv. oryzae PXO86 (Nipponbare) / mock treated leaf samples (Nipponb...	0.30	1.23	0.002
X. oryzae pv. oryzae PXO86 (Nipponbare) / X. oryzae pv. oryzae T7174 (Nipponba...	-0.05	-1.03	0.430
X. oryzae pv. oryzae PXO86 (Nipponbare) / X. oryzae pv. oryzae PXO99A (Nippon...	-0.01	-1.01	0.856
X. oryzae pv. oryzae PXO99 (Huahui 1) / untreated leaf samples (Huahui 1)	-0.02	-1.01	0.657
X. oryzae pv. oryzae PXO99 (MH63) / untreated leaf samples (MH63)	-0.00	-1.00	0.977
X. oryzae pv. oryzae PXO99A (IR24) / mock treated leaf samples (IR24)	0.20	1.16	0.113
X. oryzae pv. oryzae PXO99A (Nipponbare) / mock treated leaf samples (Nippon...	0.31	1.25	0.011
X. oryzae pv. oryzae PXO99AME5 (IR24) / mock treated leaf samples (IR24)	0.14	1.10	0.106
X. oryzae pv. oryzae PXO99AME5 (IR24) / X. oryzae pv. oryzae PXO99A (IR24	-0.06	-1.05	0.580
X. oryzae pv. oryzae PXO99AME5 (Nipponbare) / mock treated leaf samples (Nip...	0.20	1.15	0.010
X. oryzae pv. oryzae PXO99AME5 (Nipponbare) / X. oryzae pv. oryzae PXO99A (Ni...	-0.11	-1.08	0.217
X. oryzae pv. oryzae PXO99AME7 (IR24) / mock treated leaf samples (IR24)	-0.13	-1.10	0.064
X. oryzae pv. oryzae PXO99AME7 (IR24) / X. oryzae pv. oryzae PXO99A (IR24	-0.34	-1.27	0.025
X. oryzae pv. oryzae PXO99AME7 (Nipponbare) / mock treated leaf samples (Nip...	0.19	1.14	0.002
X. oryzae pv. oryzae PXO99AME7 (Nipponbare) / X. oryzae pv. oryzae PXO99A (Ni...	-0.13	-1.10	0.133
X. oryzae pv. oryzae study 3 (IR24) / X. oryzae pv. oryzae study 2 (IR24	0.22	1.17	0.023
X. oryzae pv. oryzae study 3 (IRBB5) / X. oryzae pv. oryzae study 2 (IRBB5	0.44	1.35	0.001
X. oryzae pv. oryzae study 3 (IRBB7) / X. oryzae pv. oryzae study 2 (IRBB7	0.28	1.22	0.034
X. oryzae pv. oryzae study 4 (IR24) / X. oryzae pv. oryzae study 2 (IR24	0.36	1.29	0.043
X. oryzae pv. oryzae study 4 (IRBB5) / X. oryzae pv. oryzae study 2 (IRBB5	0.42	1.34	0.005
X. oryzae pv. oryzae study 4 (IRBB7) / X. oryzae pv. oryzae study 2 (IRBB7	0.29	1.23	0.011
X. oryzae pv. oryzae study 5 (IR24) / X. oryzae pv. oryzae study 2 (IR24	0.21	1.16	0.048
X. oryzae pv. oryzae study 5 (IRBB5) / X. oryzae pv. oryzae study 2 (IRBB5	0.22	1.17	0.054
X. oryzae pv. oryzae study 5 (IRBB7) / X. oryzae pv. oryzae study 2 (IRBB7	0.06	1.05	0.569
X. oryzae pv. oryzae study 6 (IR24) / X. oryzae pv. oryzae study 2 (IR24	0.13	1.10	0.316
X. oryzae pv. oryzae study 6 (IRBB5) / X. oryzae pv. oryzae study 2 (IRBB5	0.22	1.16	0.018
X. oryzae pv. oryzae study 6 (IRBB7) / X. oryzae pv. oryzae study 2 (IRBB7	0.14	1.10	0.087
X. oryzae pv. oryzae study 7 (BAI3) / mock treated leaf samples	0.20	1.15	0.120
X. oryzae pv. oryzae study 7 (BAI3Talc) / X. oryzae pv. oryzae study 7 (BAI3	-0.06	-1.05	0.506
X. oryzae pv. oryzae study 7 (BAI3Talc) / mock treated leaf samples	0.13	1.10	0.092
X. oryzae pv. oryzae T7174 (IR24) / mock treated leaf samples (IR24)	0.11	1.08	0.339
X. oryzae pv. oryzae T7174 (IR24) / X. oryzae pv. oryzae PXO99A (IR24	-0.10	-1.07	0.502
X. oryzae pv. oryzae T7174 (Nipponbare) / mock treated leaf samples (Nipponba...	0.35	1.27	0.001
X. oryzae pv. oryzae T7174 (Nipponbare) / X. oryzae pv. oryzae PXO99A (Nipponb...	0.03	1.02	0.689
X. oryzae pv. oryzicola (24hpi) / mock treated shoot samples (24hpi)	-0.00	1.00	0.996
X. oryzae pv. oryzicola (24hpi) / X. oryzae pv. oryzae (24hpi)	-0.02	-1.01	0.894
X. oryzae pv. oryzicola (2hpi) / X. oryzae pv. oryzae (2hpi)	-0.02	-1.02	0.883
X. oryzae pv. oryzicola (2hpi) / mock treated shoot samples (2hpi)	0.08	1.06	0.392
X. oryzae pv. oryzicola (4hpi) / X. oryzae pv. oryzae (4hpi)	-0.07	-1.05	0.384
X. oryzae pv. oryzicola (4hpi) / mock treated shoot samples (4hpi)	0.02	1.01	0.748
X. oryzae pv. oryzicola (8hpi) / X. oryzae pv. oryzae (8hpi)	-0.04	-1.04	0.824
X. oryzae pv. oryzicola (8hpi) / mock treated shoot samples (8hpi)	-0.00	-1.00	0.977
X. oryzae pv. oryzicola (96hpi) / mock treated shoot samples (96hpi)	-0.07	-1.06	0.635
X. oryzae pv. oryzicola (96hpi) / X. oryzae pv. oryzae (96hpi)	-0.10	-1.08	0.496
X. oryzae pv. oryzicola study 2 (9804) / untreated flag leaf samples (9804)	-0.01	-1.02	0.948
X. oryzae pv. oryzicola study 2 (9804-Rxo1) / untreated flag leaf samples (9804-...	0.49	1.40	0.057
X. oryzae pv. oryzicola study 3 (IR24) / mock treated leaf samples (IR24)	0.19	1.14	0.030
X. oryzae pv. oryzicola study 3 (Nipponbare) / mock treated leaf samples (Nippo...	0.16	1.12	0.006
X. oryzae pv. oryzicola study 4 (Huahui 1) / untreated leaf samples (Huahui 1)	-0.14	-1.10	0.019
X. oryzae pv. oryzicola study 4 (MH63) / untreated leaf samples (MH63)	0.13	1.09	0.091
▼ Chemical			
As(V) / untreated root samples	0.27	1.21	0.015
AsO4 (Azucena) / untreated seedlings (Azucena)	0.29	1.22	0.107
AsO4 (Bala) / untreated seedlings (Bala)	0.28	1.21	0.040
Cd / untreated root samples	0.24	1.18	0.010
Cr(VI) / untreated root samples	0.44	1.36	<0.001
Pb / untreated root samples	0.03	1.02	0.286
▼ Elicitor			
ClsA / mock treated leaf samples	-0.08	-1.04	0.737
▼ Hormone			
BAP / solvent treated seedling samples	-0.03	-1.02	0.153
GA3 (Zhenshan 97) / untreated seedling samples (Zhenshan 97)	0.60	1.52	<0.001
IAA / solvent treated seedling samples	0.04	1.03	0.148
KT (Zhenshan 97) / untreated seedling samples (Zhenshan 97)	0.50	1.43	0.026
NAA (Zhenshan 97) / untreated seedling samples (Zhenshan 97)	0.45	1.38	0.030
trans-zeatin (early) / solvent treated root samples (early)	0.26	1.20	0.002
trans-zeatin (late) / solvent treated root samples (late)	0.15	1.11	0.156
trans-zeatin study 2 (early) / solvent treated leaf samples (early)	0.19	1.14	0.004
trans-zeatin study 2 (late) / solvent treated leaf samples (late)	0.10	1.07	0.020
▼ Nutrient			
Fe and P deficiency (root) / Fe + P (root)	-0.11	-1.08	0.023
Fe and P deficiency (shoot) / Fe + P (shoot)	0.11	1.08	0.252
Fe deficiency (root) / Fe and P deficiency (root)	0.39	1.31	0.001
Fe deficiency (root) / Fe + P (root)	0.29	1.22	0.001
P deficiency (root) / Fe and P deficiency (root)	-0.17	-1.13	0.032
P deficiency (root) / Fe + P (root)	-0.28	-1.21	0.012
P deficiency (shoot) / Fe + P (shoot)	0.32	1.25	0.053
P deficiency (shoot) / Fe and P deficiency (shoot)	0.21	1.15	0.056
▼ Other			
aerobic germination (27h) / anaerobic germination (27h)	0.04	1.03	0.355
aerobic germination (30h) / anaerobic germination (30h)	-0.01	-1.01	0.959
aerobic germination study 2 (12h) / aerobic germination study 2 (1h)	0.44	1.37	0.035
aerobic germination study 2 (12h) / embryo samples from dry seeds	0.49	1.41	0.024
aerobic germination study 2 (1h) / embryo samples from dry seeds	0.05	1.04	0.506
aerobic germination study 2 (24h) / aerobic germination study 2 (1h)	0.15	1.11	0.141
aerobic germination study 2 (24h) / embryo samples from dry seeds	0.20	1.15	0.063
aerobic germination study 2 (3h) / aerobic germination study 2 (1h)	0.33	1.25	0.008
aerobic germination study 2 (3h) / embryo samples from dry seeds	0.38	1.30	0.004
gravistimulation (0.5h) / untreated Zhonghua11 shoot base samples	0.18	1.14	<0.001
gravistimulation (6h) / untreated Zhonghua11 shoot base samples	0.23	1.17	0.002
gravistimulation study 2 (0.5h) / untreated Zhonghua11 shoot base samples	0.10	1.07	0.009
gravistimulation study 2 (0.5h) / gravistimulation (0.5h)	-0.09	-1.06	0.006
gravistimulation study 2 (6h) / untreated Zhonghua11 shoot base samples	0.14	1.10	0.035
gravistimulation study 2 (6h) / gravistimulation (6h)	-0.09	-1.06	0.150
root impedance (Azucena) / above layer root tip samples (Azucena)	-0.03	-1.02	0.273
root impedance (Bala) / root impedance (Azucena)	-0.04	-1.02	0.631
root impedance study 2 (Azucena) / above layer root tip samples (Azucena)	-0.08	-1.06	0.460
▼ Photoperiod			
diurnal (0:00am day 2) / diurnal (8:00am day 1)	0.25	1.19	0.251
diurnal (12:00am day 1) / diurnal (8:00am day 1)	0.20	1.15	0.122
diurnal (12:00am day 2) / diurnal (8:00am day 2)	0.36	1.29	0.081
diurnal (4:00am day 2) / diurnal (8:00am day 1)	-0.01	-1.01	0.902
diurnal (4:00pm day 1) / diurnal (8:00am day 1)	0.29	1.22	0.223
diurnal (4:00pm day 2) / diurnal (8:00am day 2)	0.42	1.34	0.189
diurnal (8:00am day 2) / diurnal (8:00am day 1)	-0.23	-1.17	0.153
diurnal (8:00pm day 1) / diurnal (8:00am day 1)	0.31	1.24	0.076
diurnal (8:00pm day 2) / diurnal (8:00am day 2)	0.84	1.78	0.009
diurnal study 2 (0:00am day 2) / diurnal study 2 (8:00am day 1)	0.25	1.18	0.294
diurnal study 2 (12:00am day 1) / diurnal study 2 (8:00am day 1)	0.19	1.13	0.377
diurnal study 2 (12:00am day 2) / diurnal study 2 (8:00am day 2)	0.58	1.50	0.033
diurnal study 2 (4:00am day 2) / diurnal study 2 (8:00am day 1)	0.09	1.06	0.674
diurnal study 2 (4:00pm day 1) / diurnal study 2 (8:00am day 1)	0.30	1.23	0.220
diurnal study 2 (4:00pm day 2) / diurnal study 2 (8:00am day 2)	0.70	1.62	0.035
diurnal study 2 (8:00am day 2) / diurnal study 2 (8:00am day 1)	-0.18	-1.14	0.465
diurnal study 2 (8:00pm day 1) / diurnal study 2 (8:00am day 1)	0.42	1.33	0.148
diurnal study 2 (8:00pm day 2) / diurnal study 2 (8:00am day 2)	0.64	1.56	0.034
▼ Stress			
anaerobic germination (12h) / anaerobic germination (1h)	0.55	1.47	0.003
anaerobic germination (24h) / anaerobic germination (1h)	0.22	1.16	0.022
anaerobic germination (27h) / anaerobic germination (1h)	0.18	1.13	0.026
anaerobic germination (30h) / anaerobic germination (1h)	0.17	1.13	0.138
anaerobic germination (3h) / anaerobic germination (1h)	0.08	1.06	0.331
anoxica / aerobic germinated coleoptile samples	-0.61	-1.53	0.047
cold / untreated seedlings (IR64)	0.03	1.02	0.277
cold study 2 (MH63) / untreated leaf samples (MH63)	-0.24	-1.18	0.115
drought / untreated seedlings (IR64)	0.09	1.06	0.025
drought study 2 (Azucena) / untreated Azucena leaf samples	-0.31	-1.23	0.006
drought study 2 (Bala) / untreated Bala leaf samples	0.19	1.14	0.030
drought study 3 (leaf) / untreated leaf samples	0.33	1.26	0.028
drought study 3 (root) / untreated root samples	-0.02	-1.01	0.462
drought study 4 (leaf) / untreated leaf samples	-0.03	-1.03	0.811
drought study 4 (root) / untreated root samples	-0.23	-1.17	<0.001
drought study 5 (leaf) / untreated leaf samples	0.05	1.04	0.471
drought study 5 (panicle) / untreated panicle samples	-0.08	-1.05	0.320
drought study 7 (IRAT109) / untreated flag leaf samples (IRAT109)	-0.55	-1.45	0.065
drought study 7 (Zhenshan 97) / untreated flag leaf samples (Zhenshan 97)	-0.32	-1.25	0.055
drought study 8 (IRAT109) / untreated flag leaf samples (IRAT109)	-0.75	-1.68	0.005
drought study 8 (Zhenshan 97) / untreated flag leaf samples (Zhenshan 97)	-0.45	-1.37	0.010
drought study 9 (IRAT109) / untreated flag leaf samples (IRAT109)	-0.51	-1.42	0.012
drought study 9 (Zhenshan 97) / untreated flag leaf samples (Zhenshan 97)	-0.46	-1.38	0.011
heat / untreated seedling samples	-0.67	-1.64	0.051
heat study 2 (MH63) / untreated leaf samples (MH63)	0.38	1.30	0.024
salt (FL478) / untreated growing point samples (FL478)	0.00	-1.01	0.994
salt (IR29) / untreated growing point samples (IR29)	0.03	1.02	0.665
salt study 2 / untreated seedlings (IR64)	-0.04	-1.03	0.445
salt study 3 (FL478) / untreated root samples (FL478)	-0.03	-1.02	0.489
salt study 3 (IR29) / untreated root samples (IR29)	0.00	1.00	0.982
salt study 4 (salt sensitive RILs) / untreated seedling samples (salt sensitive RI...	-0.30	-1.23	0.008
salt study 4 (salt tolerant RILs) / untreated seedling samples (salt tolerant RILs)	-0.14	-1.10	0.475
salt study 5 (IR63731) / untreated root samples (IR63731)	0.02	1.01	0.728
salt study 5 (Pokkali) / untreated root samples (Pokkali)	-0.01	-1.00	0.914
shift aerobic to anaerobic germination (24h+3h) / anaerobic germination (27h)	0.04	1.03	0.367
shift aerobic to anaerobic germination (24h+3h) / aerobic germination (27h)	0.01	1.00	0.902
shift aerobic to anaerobic germination (24h+6h) / aerobic germination (30h)	-0.07	-1.05	0.244
shift aerobic to anaerobic germination (24h+6h) / anaerobic germination (30h)	-0.07	-1.05	0.438
shift anaerobic to aerobic germination (24h+3h) / anaerobic germination (27h)	0.11	1.08	0.026
shift anaerobic to aerobic germination (24h+3h) / aerobic germination (27h)	0.07	1.05	0.063
shift anaerobic to aerobic germination (24h+6h) / aerobic germination (30h)	-0.00	-1.00	0.956
shift anaerobic to aerobic germination (24h+6h) / anaerobic germination (30h)	-0.01	-1.01	0.928
submergence (M202(Sub1)) / untreated shoot samples (M202(Sub1))	0.23	1.18	0.138
submergence (M202) / untreated shoot samples (M202)	0.17	1.12	0.072
▼ Temperature			
31°C (Minghui 63) / untreated calli samples (Minghui 63)	-0.25	-1.19	0.018
31°C (Zhenshan 97) / untreated calli samples (Zhenshan 97)	-0.17	-1.12	0.021
93-11 / Nipponbare	0.02	1.02	0.640
93-11 / Nipponbare	0.20	1.15	0.013
▼ 9804			
9804-Rxo1 / 9804	-0.35	-1.28	0.205
9804-Rxo1 / 9804	0.15	1.12	0.441
▼ Asominori			
CSSL50-1 / Asominori	0.01	1.01	0.865
Bala / Azucena	-0.20	-1.15	0.015
Bala / Azucena	0.29	1.22	0.011
▼ Bengal			
ScFv / Bengal	0.05	1.03	0.609
▼ Estrela A			
Estrela mut. / Estrela A	-0.10	-1.07	0.030
▼ Fujiminori			
Fukei 71 / Fujiminori	0.06	1.04	0.110
▼ Hwayoung			
chr729 / Hwayoung	-0.10	-1.08	0.517
▼ IR24			
IR24 / Nipponbare	0.32	1.25	0.040
IR24 / Nipponbare	0.19	1.14	0.015
IR24 / Nipponbare	0.08	1.06	0.504
IR24 / Nipponbare	-0.13	-1.09	0.023
IR24 / Nipponbare	0.13	1.10	0.114
IR24 / Nipponbare	-0.05	-1.03	0.671
IR24 / Nipponbare	0.24	1.18	0.025
IR24 / Nipponbare	0.23	1.17	0.008
IRBB5 / IR24	-0.03	-1.02	0.636
IRBB5 / IR24	0.18	1.13	0.028
IRBB5 / IR24	0.03	1.02	0.841
IRBB5 / IR24	-0.01	-1.01	0.880
IRBB5 / IR24	0.05	1.03	0.658
IRBB7 / IRBB5	0.06	1.04	0.289
IRBB7 / IRBB5	-0.09	-1.06	0.360
IRBB7 / IRBB5	-0.07	-1.05	0.437
IRBB7 / IRBB5	-0.10	-1.07	0.445
IRBB7 / IRBB5	-0.02	-1.01	0.810
IRBB7 / IR24	0.03	1.02	0.579
IRBB7 / IR24	0.09	1.07	0.401
IRBB7 / IR24	-0.04	-1.03	0.793
IRBB7 / IR24	-0.11	-1.08	0.359
IRBB7 / IR24	0.04	1.02	0.772
▼ IR29			
FL478 / IR29	-0.03	-1.01	0.881
FL478 / IR29	0.07	1.05	0.175
FL478 / IR29	0.04	1.03	0.450
IRAT109 / Zhenshan 97	-0.06	-1.04	0.466
IRAT109 / Zhenshan 97	-0.29	-1.22	0.197
IRAT109 / Zhenshan 97	-0.36	-1.29	0.001
IRAT109 / Zhenshan 97	-0.11	-1.08	0.026
▼ Kitaake			
PSARK::IPT / Kitaake	0.01	-1.00	0.966
▼ KMR3			
IL50-7 / KMR3	0.10	1.07	0.372
IL50-7 / KMR3	-0.23	-1.17	0.030
▼ M202			
M202(Sub1) / M202	-0.16	-1.12	0.016
M202(Sub1) / M202	-0.09	-1.06	0.478
▼ MH63			
Huahui 1 / MH63	0.07	1.05	0.245
Huahui 1 / MH63	0.17	1.13	0.236
Huahui 1 / MH63	0.15	1.11	0.128
Huahui 1 / MH63	0.25	1.18	0.016
Huahui 1 / MH63	-0.01	-1.01	0.787
Huahui 1 / MH63	0.23	1.17	0.073
Huahui 1 / MH63	-0.02	-1.01	0.552
Huahui 1 / MH63	0.10	1.07	0.103
Huahui 1 / MH63	-0.03	-1.02	0.662
MH63 / Zhenshan 97	0.57	1.49	0.015
OsSRT1RNAi / MH63	-0.17	-1.12	0.029
▼ Mudanjiang 8			
OsWRKY13-ox / Mudanjiang 8	-0.10	-1.07	0.281
▼ Nipponbare			
coi1-13 / Nipponbare	0.16	1.12	0.049
Nip.100Gy / Nipponbare	-0.28	-1.21	0.071
Nip.GM / Nipponbare	0.04	1.03	0.266
Nipponbare / IAC 165	0.22	1.17	0.144
Nipponbare / IAC 165	0.06	1.04	0.355
Nipponbare / IAC 165	0.14	1.10	0.065
Nipponbare / IAC 165	0.13	1.09	0.150
Nipponbare / IAC 165	-0.03	-1.02	0.467
Nipponbare / IAC 165	0.04	1.03	0.400
OsRR6-ox / Nipponbare/ev	-0.21	-1.15	0.009
RK16331-13 / FOX3	-0.02	-1.00	0.934
▼ not specified strain			
▼ Gui-630 x Taiwanjing			
lemmata / Gui-630 x Taiwanjing	0.02	1.01	0.865
IR63731 / Pokkali	-0.05	-1.04	0.136
IR63731 / Pokkali	-0.03	-1.02	0.754
KT95-418 / WuYun3	0.15	1.12	0.423
KT95-418 / WuYun3	-0.02	-1.01	0.850
▼ MI 48 x CSR 27			
salt tolerant RILs / salt sensitive RILs	-0.10	-1.07	0.009
salt tolerant RILs / salt sensitive RILs	0.06	1.05	0.759
▼ Zhenshan 97 x Minghui 63			
RIL001 / Zhenshan 97	0.19	1.14	0.108
RIL001 / MH63	-0.39	-1.31	0.160
RIL004 / Zhenshan 97	0.15	1.11	0.335
RIL004 / MH63	-0.42	-1.34	0.059
RIL005 / Zhenshan 97	-0.21	-1.16	0.094
RIL005 / MH63	-0.79	-1.73	0.047
RIL006 / Zhenshan 97	0.28	1.21	0.016
RIL006 / MH63	-0.29	-1.23	0.086
RIL007 / Zhenshan 97	0.05	1.03	0.637
RIL007 / MH63	-0.53	-1.44	0.027
RIL009 / Zhenshan 97	0.10	1.07	0.191
RIL009 / MH63	-0.47	-1.39	0.117
RIL012 / Zhenshan 97	-0.06	-1.04	0.702
RIL012 / MH63	-0.64	-1.55	0.026
RIL013 / Zhenshan 97	-0.43	-1.28	0.450
RIL013 / MH63	-1.00	-1.91	0.070
RIL014 / Zhenshan 97	-0.13	-1.09	0.505
RIL014 / MH63	-0.70	-1.62	0.024
RIL015 / Zhenshan 97	-0.12	-1.07	0.668
RIL015 / MH63	-0.69	-1.60	0.047
RIL016 / Zhenshan 97	0.14	1.10	0.256
RIL016 / MH63	-0.43	-1.35	0.046
RIL017 / Zhenshan 97	0.24	1.18	0.083
RIL017 / MH63	-0.33	-1.26	0.204
RIL018 / Zhenshan 97	0.13	1.10	0.152
RIL018 / MH63	-0.44	-1.36	0.130
RIL019 / Zhenshan 97	-0.12	-1.07	0.648
RIL019 / MH63	-0.69	-1.60	0.040
RIL020 / Zhenshan 97	0.03	1.03	0.797
RIL020 / MH63	-0.54	-1.46	0.030
RIL022 / Zhenshan 97	0.20	1.15	0.100
RIL022 / MH63	-0.37	-1.30	0.171
RIL023 / Zhenshan 97	0.12	1.09	0.169
RIL023 / MH63	-0.45	-1.38	0.123
RIL026 / Zhenshan 97	0.11	1.08	0.179
RIL026 / MH63	-0.46	-1.38	0.120
RIL027 / Zhenshan 97	-0.13	-1.09	0.153
RIL027 / MH63	-0.70	-1.63	0.058
RIL030 / Zhenshan 97	0.04	1.03	0.437
RIL030 / MH63	-0.53	-1.45	0.094
RIL039 / Zhenshan 97	0.10	1.08	0.512
RIL039 / MH63	-0.47	-1.39	0.050
RIL042 / Zhenshan 97	-0.04	-1.02	0.836
RIL042 / MH63	-0.61	-1.52	0.036
RIL047 / Zhenshan 97	0.09	1.07	0.459
RIL047 / MH63	-0.48	-1.40	0.037
RIL048 / Zhenshan 97	0.13	1.09	0.135
RIL048 / MH63	-0.44	-1.37	0.034
RIL053 / Zhenshan 97	0.30	1.24	0.067
RIL053 / MH63	-0.27	-1.21	0.268
RIL054 / Zhenshan 97	-0.15	-1.10	0.439
RIL054 / MH63	-0.72	-1.65	0.021
RIL059 / Zhenshan 97	0.24	1.19	0.124
RIL059 / MH63	-0.33	-1.26	0.091
RIL062 / Zhenshan 97	0.20	1.15	0.102
RIL062 / MH63	-0.38	-1.30	0.167
RIL066 / Zhenshan 97	-0.18	-1.10	0.657
RIL066 / MH63	-0.75	-1.64	0.077
RIL068 / Zhenshan 97	0.05	1.05	0.819
RIL068 / MH63	-0.52	-1.42	0.074
RIL071 / Zhenshan 97	-0.43	-1.34	0.048
RIL071 / MH63	-1.00	-2.00	0.030
RIL072 / Zhenshan 97	0.15	1.11	0.443
RIL072 / MH63	-0.43	-1.34	0.078
RIL077 / Zhenshan 97	0.16	1.12	0.127
RIL077 / MH63	-0.41	-1.34	0.143
RIL080 / Zhenshan 97	0.11	1.09	0.605
RIL080 / MH63	-0.46	-1.37	0.077
RIL081 / Zhenshan 97	0.05	1.04	0.792
RIL081 / MH63	-0.52	-1.43	0.055
RIL082 / Zhenshan 97	0.04	1.03	0.840
RIL082 / MH63	-0.54	-1.45	0.046
RIL083 / Zhenshan 97	0.19	1.14	0.107
RIL083 / MH63	-0.39	-1.31	0.161
RIL084 / Zhenshan 97	-0.36	-1.29	0.056
RIL084 / MH63	-0.94	-1.92	0.034
RIL086 / Zhenshan 97	0.00	1.00	0.930
RIL086 / MH63	-0.57	-1.49	0.084
RIL087 / Zhenshan 97	0.06	1.05	0.602
RIL087 / MH63	-0.51	-1.42	0.034
RIL089 / Zhenshan 97	-0.05	-1.04	0.395
RIL089 / MH63	-0.63	-1.55	0.013
RIL090 / Zhenshan 97	0.04	1.04	0.878
RIL090 / MH63	-0.53	-1.43	0.082
RIL095 / Zhenshan 97	0.35	1.27	0.059
RIL095 / MH63	-0.23	-1.17	0.327
RIL099 / Zhenshan 97	0.11	1.08	0.038
RIL099 / MH63	-0.46	-1.38	0.028
RIL101 / Zhenshan 97	0.18	1.13	0.114
RIL101 / MH63	-0.40	-1.32	0.155
RIL103 / Zhenshan 97	0.10	1.07	0.515
RIL103 / MH63	-0.48	-1.39	0.047
RIL105 / Zhenshan 97	0.14	1.10	0.220
RIL105 / MH63	-0.43	-1.35	0.043
RIL106 / Zhenshan 97	0.04	1.03	0.704
RIL106 / MH63	-0.53	-1.45	0.026
RIL109 / Zhenshan 97	0.13	1.09	0.153
RIL109 / MH63	-0.44	-1.36	0.129
RIL111 / Zhenshan 97	0.18	1.14	0.110
RIL111 / MH63	-0.39	-1.32	0.158
RIL112 / Zhenshan 97	-0.11	-1.05	0.773
RIL112 / MH63	-0.69	-1.57	0.092
RIL114 / Zhenshan 97	-0.10	-1.05	0.723
RIL114 / MH63	-0.67	-1.57	0.050
RIL118 / Zhenshan 97	0.36	1.28	0.057
RIL118 / MH63	-0.21	-1.16	0.350
RIL123 / Zhenshan 97	0.18	1.13	0.115
RIL123 / MH63	-0.40	-1.32	0.154
RIL125 / Zhenshan 97	-0.02	1.01	0.963
RIL125 / MH63	-0.59	-1.48	0.106
RIL129 / Zhenshan 97	-0.50	-1.41	0.041
RIL129 / MH63	-1.07	-2.11	0.026
RIL131 / Zhenshan 97	-0.30	-1.18	0.570
RIL131 / MH63	-0.88	-1.76	0.090
RIL133 / Zhenshan 97	-0.12	-1.09	0.162
RIL133 / MH63	-0.70	-1.63	0.059
RIL134 / Zhenshan 97	-0.14	-1.08	0.689
RIL134 / MH63	-0.71	-1.61	0.068
RIL137 / Zhenshan 97	0.11	1.08	0.379
RIL137 / MH63	-0.46	-1.38	0.043
RIL139 / Zhenshan 97	-0.35	-1.28	0.058
RIL139 / MH63	-0.92	-1.91	0.035
RIL140 / Zhenshan 97	-0.27	-1.17	0.509
RIL140 / MH63	-0.84	-1.75	0.055
RIL141 / Zhenshan 97	0.13	1.09	0.154
RIL141 / MH63	-0.44	-1.36	0.129
RIL145 / Zhenshan 97	-0.01	1.00	0.968
RIL145 / MH63	-0.58	-1.49	0.037
RIL147 / Zhenshan 97	-0.31	-1.24	0.016
RIL147 / MH63	-0.88	-1.85	0.005
RIL149 / Zhenshan 97	-0.01	-1.01	0.824
RIL149 / MH63	-0.58	-1.50	0.081
RIL150 / Zhenshan 97	-0.34	-1.26	0.060
RIL150 / MH63	-0.91	-1.89	0.036
RIL151 / Zhenshan 97	0.15	1.11	0.132
RIL151 / MH63	-0.42	-1.34	0.140
RIL152 / Zhenshan 97	0.24	1.18	0.086
RIL152 / MH63	-0.34	-1.27	0.196
RIL154 / Zhenshan 97	-0.11	-1.08	0.187
RIL154 / MH63	-0.68	-1.61	0.062
RIL155 / Zhenshan 97	-0.01	-1.00	0.952
RIL155 / MH63	-0.59	-1.49	0.045
RIL156 / Zhenshan 97	0.10	1.08	0.290
RIL156 / MH63	-0.47	-1.39	0.034
RIL159 / Zhenshan 97	0.22	1.17	0.015
RIL159 / MH63	-0.35	-1.28	0.056
RIL160 / Zhenshan 97	0.01	1.01	0.951
RIL160 / MH63	-0.56	-1.48	0.035
RIL161 / Zhenshan 97	-0.00	1.01	0.999
RIL161 / MH63	-0.57	-1.48	0.058
RIL162 / Zhenshan 97	-0.42	-1.30	0.362
RIL162 / MH63	-1.00	-1.94	0.042
RIL163 / Zhenshan 97	-0.02	-1.01	0.915
RIL163 / MH63	-0.59	-1.50	0.033
RIL164 / Zhenshan 97	0.18	1.14	0.110
RIL164 / MH63	-0.39	-1.31	0.159
RIL165 / Zhenshan 97	0.17	1.12	0.120
RIL165 / MH63	-0.40	-1.33	0.149
RIL167 / Zhenshan 97	-0.57	-1.49	0.035
RIL167 / MH63	-1.15	-2.22	0.023
RIL169 / Zhenshan 97	0.03	1.02	0.681
RIL169 / MH63	-0.54	-1.46	0.021
RIL173 / Zhenshan 97	0.02	1.02	0.849
RIL173 / MH63	-0.55	-1.47	0.027
RIL175 / Zhenshan 97	0.10	1.07	0.206
RIL175 / MH63	-0.48	-1.40	0.114
RIL178 / Zhenshan 97	0.25	1.19	0.083
RIL178 / MH63	-0.33	-1.26	0.205
RIL180 / Zhenshan 97	0.01	1.01	0.976
RIL180 / MH63	-0.57	-1.47	0.052
RIL183 / Zhenshan 97	0.05	1.03	0.679
RIL183 / MH63	-0.53	-1.44	0.029
RIL184 / Zhenshan 97	0.06	1.04	0.326
RIL184 / MH63	-0.52	-1.44	0.100
RIL185 / Zhenshan 97	-0.29	-1.22	0.070
RIL185 / MH63	-0.86	-1.83	0.039
RIL186 / Zhenshan 97	0.03	1.02	0.857
RIL186 / MH63	-0.55	-1.46	0.034
RIL187 / Zhenshan 97	-0.09	-1.04	0.799
RIL187 / MH63	-0.66	-1.55	0.077
RIL191 / Zhenshan 97	0.07	1.06	0.771
RIL191 / MH63	-0.50	-1.40	0.085
RIL192 / Zhenshan 97	0.17	1.13	0.117
RIL192 / MH63	-0.40	-1.32	0.152
RIL193 / Zhenshan 97	0.14	1.10	0.147
RIL193 / MH63	-0.44	-1.36	0.132
RIL195 / Zhenshan 97	-0.16	-1.12	0.056
RIL195 / MH63	-0.74	-1.67	0.008
RIL197 / Zhenshan 97	-0.23	-1.16	0.423
RIL197 / MH63	-0.80	-1.73	0.030
RIL198 / Zhenshan 97	-0.18	-1.10	0.667
RIL198 / MH63	-0.76	-1.64	0.084
RIL199 / Zhenshan 97	-0.18	-1.13	0.357
RIL199 / MH63	-0.76	-1.69	0.018
RIL203 / Zhenshan 97	-0.08	-1.05	0.669
RIL203 / MH63	-0.66	-1.57	0.031
RIL204 / Zhenshan 97	0.05	1.03	0.392
RIL204 / MH63	-0.53	-1.45	0.096
RIL205 / Zhenshan 97	0.14	1.11	0.396
RIL205 / MH63	-0.43	-1.34	0.066
RIL206 / Zhenshan 97	-0.26	-1.20	0.017
RIL206 / MH63	-0.83	-1.79	0.006
RIL209 / Zhenshan 97	0.06	1.04	0.302
RIL209 / MH63	-0.51	-1.43	0.102
RIL210 / Zhenshan 97	-0.01	-1.00	0.967
RIL210 / MH63	-0.58	-1.49	0.032
RIL215 / Zhenshan 97	-0.52	-1.43	0.039
RIL215 / MH63	-1.09	-2.14	0.025
RIL216 / Zhenshan 97	0.20	1.15	0.099
RIL216 / MH63	-0.37	-1.30	0.172
RIL219 / Zhenshan 97	0.27	1.20	0.076
RIL219 / MH63	-0.31	-1.24	0.224
RIL232 / Zhenshan 97	-0.10	-1.06	0.722
RIL232 / MH63	-0.67	-1.58	0.053
RIL236 / Zhenshan 97	0.17	1.13	0.118
RIL236 / MH63	-0.40	-1.33	0.150
RIL238 / Zhenshan 97	0.09	1.06	0.219
RIL238 / MH63	-0.48	-1.40	0.111
RIL241 / Zhenshan 97	0.09	1.06	0.241
RIL241 / MH63	-0.48	-1.40	0.028
Pusa 1266 / Pusa Basmati 1	0.07	1.05	0.182
▼ Pusa Basmati 1			
CODA-22 / Pusa Basmati 1	0.04	1.03	0.383
CODA-4 / Pusa Basmati 1	-0.07	-1.05	0.082
Rathu Heenati / Taichung Native-1	0.03	1.02	0.562
Rathu Heenati / Taichung Native-1	-0.04	-1.03	0.720
Rathu Heenati / Taichung Native-1	0.13	1.09	0.176
▼ Senia			
Senia-afp / Senia	-0.15	-1.11	0.017
▼ Taichung 65			
gid1-3 / Taichung 65	0.21	1.15	0.011
gid2-1 / Taichung 65	-0.03	-1.02	0.625
slr1 (k1001) / Taichung 65	0.06	1.04	0.089
▼ Taipei 309			
TP-Pi54-15 / Taipei 309	0.18	1.13	0.173
TP-Pi54-15 / Taipei 309	0.22	1.16	0.243
▼ Xiushui 11			
KMD / Xiushui 11	-0.08	-1.06	0.110
Zhenshan 97 / Nipponbare	0.02	1.01	0.892
Zhenshan 97 / Nipponbare	0.25	1.19	0.266
Zhenshan 97 / Nipponbare	0.22	1.16	0.547
Zhenshan 97 / Nipponbare	0.04	1.03	0.429
Zhenshan 97 / Nipponbare	-0.05	-1.03	0.709
Zhenshan 97 / MH63	-0.08	-1.06	0.200
Zhenshan 97 / MH63	0.00	1.00	0.570
Zhenshan 97 / MH63	-0.20	-1.14	0.399
Zhenshan 97 / MH63	-0.07	-1.05	0.064
Zhenshan 97 / MH63	0.11	1.08	0.202
Zhenshan 97 / MH63	0.01	1.00	0.918
Zhenshan 97 / MH63	-0.10	-1.07	0.267
Zhenshan 97 / MH63	-0.09	-1.06	0.307
Zhenshan 97 / MH63	-0.11	-1.08	0.187
Zhenshan 97 / MH63	0.03	1.02	0.848
Zhenshan 97 / MH63	0.13	1.10	0.608
Zhenshan 97 / MH63	0.24	1.17	0.279
Zhenshan 97 / MH63	0.26	1.21	0.623
Zhenshan 97 / MH63	0.03	1.02	N/A
Zhenshan 97 / MH63	0.05	1.03	0.620
Zhenshan 97 / MH63	-0.55	-1.45	0.173
Zhenshan 97 / MH63	-0.38	-1.31	0.030
Zhenshan 97 / MH63	0.00	1.00	0.011
Zhenshan 97 / MH63	-0.55	-1.46	0.007
Zhenshan 97 / MH63	-0.54	-1.46	0.025
Zhenshan 97 / MH63	-0.51	-1.42	0.239
Zhenshan 97 / MH63	-0.16	-1.11	0.486
Zhenshan 97 / MH63	-0.18	-1.15	0.567
Zhenshan 97 / MH63	-0.13	-1.09	0.216
Zhenshan 97 / MH63	0.69	1.63	0.072
Zhenshan 97 / MH63	-0.07	-1.05	0.071
Zhenshan 97 / MH63	-0.13	-1.09	0.314
Zhenshan 97 / MH63	-0.02	-1.01	0.869
Zhenshan 97 / MH63	-0.07	-1.05	0.430
Zhenshan 97 / MH63	-0.10	-1.06	0.664
Zhenshan 97 / MH63	-0.07	-1.05	0.563
▼ Zhonghua11			
abl1 / Zhonghua11	-0.01	-1.01	0.601
dst / Zhonghua11	0.02	1.02	0.508
gif1 / Zhonghua11	0.16	1.12	0.174
lc1 / Zhonghua11	0.06	1.05	0.732
miR156 OX / Zhonghua11	0.11	1.08	0.473
S58S / Zhonghua11	0.11	1.08	0.163
S59R / Zhonghua11	0.34	1.27	0.001
▼ Zhongxian 3037			
phoenix (pho) / Zhongxian 3037	-0.34	-1.27	0.464

< -4 | -3 | -2 | -1 | 0 | 1 | 2 | 3 | 4 >
