# Supplementary material for: Rice UBC13, a candidate housekeeping gene, is required for K63-linked polyubiquitination and tolerance to DNA damage
Source: Rice (N Y). 2012 Sep 8;5:24. doi: 10.1186/1939-8433-5-24 (PMC5520843; doi:10.1186/1939-8433-5-24)
Supplement: Supplementary file 4 — Additional file 4:Table S1. Saccharomyces cerevisiae strains. (DOCX 51 KB) [file 12284_2012_18_MOESM4_ESM.docx]

**Supplemental materials**

**Supplemental Table S1** *Saccharomyces cerevisiae* strains

| **Strain** | **Genotype** | **Source** |
| --- | --- | --- |
| PJ69-4A | *MAT****a*** *trp1-901 leu2-3,112 ura3-52 his3-200 gal4∆ gal80∆*  *MET2::GAL7-lacZ LYS2::GAL1-HIS3 GAL2-ADE2* | P. James |
| HK578-10D | *MATa ade2-1 can1-100 his3-11,15 leu2-3,112 trp1-1 ura3-1* | H. Klein |
| WXY904 | HK578-10D with *ubc13∆::HIS3* | This study |
| DBY747 | *MAT****a*** *his3-1 leu2-3, 112 trp1-289 ura3-52* | D. Botstein |
| WXY849 | DBY747 with *ubc13∆::HIS3* | This study |

**Supplemental figure legends**

**Fig. S1** Phylogenetic analysis of selected ubiquitin conjugating enzyme (E2) family proteins based on the alignment of 59 protein sequences from 7 species (*S. cerevisiae, A. thaliana, O. sativa, D. melanogaster, C. elegans, H. sapiens, D. rerio*). The circled clade represents Ubc13s derived from NP_010377.1 (*S. cerevisiae*), NP_564011.1 and NP_849902.2 (*A. thaliana*), NP_001043834.1 (*O. sativa*), NP_001162752.1 (*D. melanogaster*), NP_500272.2 (*C. elegans*), NP_003339.1 (*H. sapiens*), NP_998651.1 (*D. rerio*). Note that the mouse and human Ubc13 sequences are identical (data not shown). Five other human Ubcs (UBE2D, UBE2E, UBE2J, UBE2K and UBE2T) that most closely related to Ubc13 in sequence and their orthologs in the above species were retrived for sequence alignment. The phylogenetic tree was drawn by using a MEGA 5.05 program.

**Fig. S2** Quantitative analysis of *OsUBC13* (LOC_Os01g48280) expression in rice tissues. Samples were taken from different rice tissues as indicated in the left column and relative transcript levels in each tissue-specific transcriptome were determined by microarray analysis. The data is retrieved from Genevestigator ([www.genevestigator.com](http://www.genevestigator.com)).

**Fig. S3** Quantitative analysis of *OsUBC13* (LOC_Os01g48280) expression in rice under different stress conditions. Samples were taken from rice tissues after the plants were treated as indicated in the left column. Relative expression levels were determined by microarray analysis. The resulting data were compiled and retrieved from Genevestigator ([www.genevestigator.com](http://www.genevestigator.com)), and displayed in log2 scale in the middle column. The actual fold change values upon stress treatment are given in the right column.
